# Three distinct mechanisms of long-distance modulation of gene expression in yeast

**DOI:** 10.1371/journal.pgen.1006736

**Published:** 2017-04-20

**Authors:** Manyu Du, Qian Zhang, Lu Bai

**Affiliations:** 1 Department of Biochemistry and Molecular Biology, the Pennsylvania State University, University Park, State College, PA, United States of America; 2 Center for Eukaryotic Gene Regulation, the Pennsylvania State University, University Park, PA, State College, United States of America; 3 Department of Physics, the Pennsylvania State University, University Park, State College, PA, United States of America; Netherlands Cancer Institute, NETHERLANDS

## Abstract

Recent Hi-C measurements have revealed numerous intra- and inter-chromosomal interactions in various eukaryotic cells. To what extent these interactions regulate gene expression is not clear. This question is particularly intriguing in budding yeast because it has extensive long-distance chromosomal interactions but few cases of gene regulation over-a-distance. Here, we developed a medium-throughput assay to screen for functional long-distance interactions that affect the average expression level of a reporter gene as well as its cell-to-cell variability (noise). We ectopically inserted an insulated *MET3* promoter (*MET3pr*) flanked by ~1kb invariable sequences into thousands of genomic loci, allowing it to make contacts with different parts of the genome, and assayed the *MET3pr* activity in single cells. Changes of *MET3pr* activity in this case necessarily involve mechanisms that function over a distance. *MET3pr* has similar activities at most locations. However, at some locations, they deviate from the norm and exhibit three distinct patterns including low expression / high noise, low expression / low noise, and high expression / low noise. We provided evidence that these three patterns of *MET3pr* expression are caused by Sir2-mediated silencing, transcriptional interference, and 3D clustering. The clustering also occurs in the native genome and enhances the transcription of endogenous Met4-targeted genes. Overall, our results demonstrate that a small fraction of long-distance chromosomal interactions can affect gene expression in yeast.

## Author summary

Eukaryotic transcription occurs within the nucleus where DNA is packaged into high order chromosome structures. Some long-distance chromosomal interactions play an important role in gene regulation in higher eukaryotic species, such as mouse and human. In budding yeast, gene expression is traditionally thought to be regulated over short distances because the upstream regulatory sequences (URSs) are usually located close to the core promoters. However, recent chromosome conformation capture experiments have detected numerous long-distance chromosomal interactions in the yeast genome. The function of these interactions in gene regulation remains unclear. Here, we developed a new assay to screen for long-distance interactions that affect the activity of a reporter gene. We found three regulatory mechanisms that act from a distance: silencing, transcriptional interference, and 3D clustering, which alter expression level of the reporter gene as well as its cell-to-cell variability. Our results demonstrate that transcription in budding yeast, similar to transcription in higher eukaryotes, can be regulated over long distances. We anticipate our assay can be used as a general platform to screen for functional long-distance chromosomal interactions that affect gene expression.

## Introduction

Cell proliferation and differentiation depend on rigorously controlled gene activities. Gene regulation is best understood at the level of linear organization of the genome, including the primary DNA sequences and arrays of closely associated regulatory proteins. The three-dimensional (3D) organization of chromosomes also plays an important role in gene regulation [1–3]. Elucidating the regulatory functions of higher order chromatin configuration is a critical component towards the fundamental understanding of eukaryotic gene regulation.

Long-distance gene regulation is best elucidated in some specific genomic loci in multi-cellular organisms, such as the locus control region of the murine β-globin genes [4–7]. With the recent development of Chromosome Conformation Capture technique (3C) and its derivatives (4C, Hi-C, etc.), numerous intra- and inter-chromosomal interactions have been detected in different model organisms [8–11]. These interaction patterns can change with cell types, developmental stages, and environmental stress [12–15]. Some of the long-distance interactions were confirmed to have functional roles. However, in general, to what extent these interactions regulate gene expression is not clear.

Traditionally, budding yeast has not been considered as a good model for gene regulation over long distance because the upstream activating sequences (UASs) tend to be adjacent to the target genes. Consistent with this idea, artificial displacement of *GAL1* UAS away from the TATA box eliminated its activity [16]. There are only several cases in which long-distance interactions have been proposed to regulate gene expression in yeast, including promoter-terminator looping [17, 18] and inter-allelic interactions between homologous genes [19, 20]. Nevertheless, the Hi-C experiment in haploid yeast cells revealed extensive long-distance interactions among the chromosomes [11, 14]. Statistical analysis of the Hi-C data showed that co-regulated yeast loci tend to cluster [21, 22], and physically proximal genes tend to co-express [23, 24]. These studies suggest a role of long-distance chromosomal interactions in gene regulation, although direct evidence is lacking.

Other than affecting the average level of gene expression among a population, long-distance interactions may also affect its cell-to-cell variability (noise). The amplitude of gene expression noise is determined by the underlying regulatory mechanism [25–27]. Intuitively, since chromosome organization can be highly dynamic among single cells [28], it may increase variation in gene expression. In one example, an artificial long-distance activation system consisting of a mouse β-globin gene and a human Locus Control Region showed high expression noise [29]. The generality of this conclusion is unclear.

In this work, we set up an experimental scheme to screen for long-distance chromosomal interactions that affect the average level and noise of gene expression. A promoter flanked by invariable sequences of more than 1kb in length is inserted ectopically at thousands of genomic loci, allowing it to make contacts with different parts of the genome. A change in promoter activity in this case would necessarily involve mechanisms that function over a distance. In a small fraction of genomic locations (30 out of 1327), we observed modulations of the promoter activity with three distinct patterns. We then thoroughly investigated the regulatory mechanisms that cause these changes. We showed that Sir-dependent silencing and transcriptional interference can repress gene expression to a comparable extent, but cause different levels of noise. We also found evidence that the reporter gene can acquire higher activity by clustering with a subset of co-regulated genes. The latter mechanism is also used in the wild-type yeast to enhance the transcription of native genes.

## Results

### Construction of a novel yeast strain library to probe long-distance regulation

**Fig 1:**
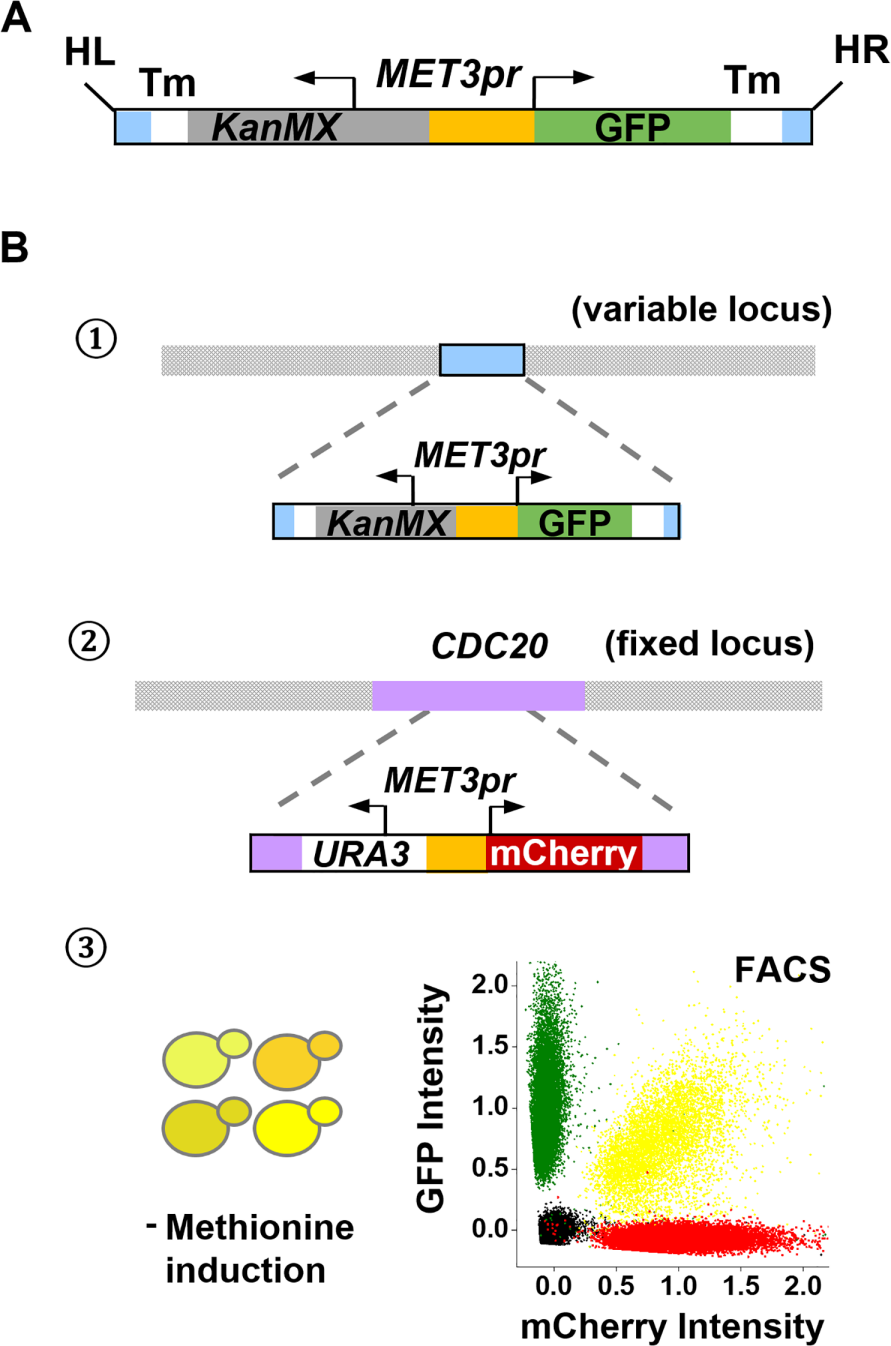
Construct of the strain library for screening long-distance regulation. **A)** Reporter gene cassette containing *MET3pr* driving GFP. Tm: Terminator. HL and HR: mTn homolog sequences. *MET3pr* is located in the middle of the cassette so that it is far from the local sequences at the integration sites. **B)** Procedure of library construction. *MET3pr-*GFP was inserted into the mTn site, and *MET3pr*-mCherry was then inserted into the *CDC20* locus. Library strains were grown under inductive conditions (no methionine), and the steady-state level of GFP and mCherry were measured in single cells using flow cytometry. Typical processed FACS data (yellow) are shown along with background control (black) and GFP only (green) / mCherry only (red) controls.

We reasoned that we want a reporter whose activity is high enough to allow accurate measurement, but not too high to potentially mask the long-distance effects. We therefore chose *MET3* promoter (*MET3pr*) driving GFP as our reporter (Fig 1A). *MET3pr* is an inducible promoter that has mild activity when induced by the depletion of methionine in the media [30]. To enable PCR-based analyses of the promoter without the interference from the endogenous *MET3pr*, we used the *MET3pr* from *S*. *kudriavzevii* in our construct. These two *MET3prs* have similar induction kinetics and steady-state expression levels (S1A and S1B Fig). They share ~50% of sequence homology and can be clearly differentiated in PCR with certain primers (S1C Fig). To selectively detect long-distance effect, we embedded the *MET3pr* in the middle of a ~3kb cassette, so that it is more than 1kb away from the variable chromosomal context at the integration site. Both edges of the cassette are flanked by terminator sequences, which should prevent (or at least reduce) invasive transcription elongation. These designs distinguish our study from previous position-dependent gene expression studies in yeast [31, 32], where the promoter of the reporter gene was placed immediately downstream an endogenous promoter, and the change in the reporter activity is more likely to reflect local instead of long-distance regulation.

To increase the chance of detecting long-distance chromosomal interactions that play a role in gene regulation, we put the reporter gene at highly dispersed genomic locations to explore different chromosomal interactions. We took advantage of the commercially available yeast insertion library, which contains more than 2000 heterozygous diploid strains each with an mTn sequence at a unique genomic locus [33] (S2 Fig). The initial mTn insertion was carried out by a transposition reaction in *E*. *coli* [34], and therefore the insertion sites are not influenced by the chromatin structure in yeast. We added the mTn sequences to the reporter cassette so that it can be integrated at the mTn loci through homologous recombination. Because the integrated reporter disrupts many open reading frames (ORFs), we integrated a *MET3pr*–mCherry control into a fixed locus to distinguish the regulatory effect specific to GFP from the global effect due to the loss of a resident gene (Fig 1B; Methods).

In total, we have constructed 1327 strains, each with a GFP reporter inserted at a unique genomic locus and mCherry at a fixed locus (see S1 and S2 Tables for strain list and expression data). We induced the strains in methionine-free synthetic media for 5–6 hours to reach the steady-state of *MET3pr* activation (S1A Fig) and measured the fluorescent intensity with flow cytometry (FACS), which allows us to evaluate the reporter gene expression in single cells (Fig 1B; Methods).

### GFP outliers show three distinct expression profiles

**Fig 2:**
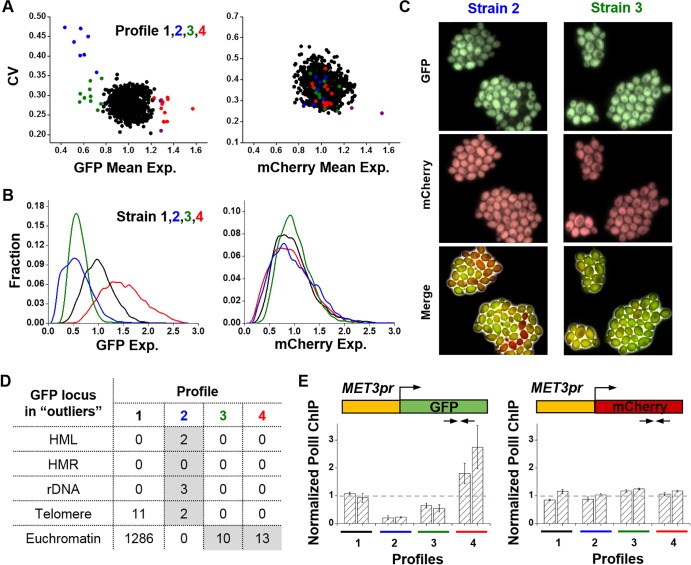
Expression profiles of the library strains. **A)** Noise versus average expression level for all strains. Each dot shows the average GFP / mCherry fluorescence level (x axis) and the CV (y axis) in one strain. In both panels, the colored dots represent the GFP outliers in profile 2 (blue), 3 (green), and 4 (red). The purple dots represent outliers in both GFP and mCherry expression. **B)** GFP (left) and mCherry (right) FACS data in four example strains, one from each profile. Strain 2, 3, and 4 are GFP outliers. Stain 2 has the GFP reporter inserted in telomere, and GFP in strain 3 and 4 are localized in apparent euchromatic regions (*SEG2* and *CWC23*). **C)** Fluorescent images of strain 2 and 3 in (B) after 5 hours of induction. These images confirm that strain 2 has higher cell-to-cell variation in GFP expression, but not in mCherry. **D)** GFP insertion sites in each profile. All the strains in profile 2 have *MET3pr*-GFP in known silencing loci (HML, rDNA, and telomere). In striking contrast, the outliers in profile 3 and 4 all have *MET3pr*-GFP located in euchromatic regions. **E)** Pol II ChIP over the GFP and mCherry ORFs in eight strains, two from each profile. The arrows mark the locations of the PCR probes. All the ChIP signals were normalized by that in the profile 1 strains. The error bars represent the standard errors among three biological replicates (the same as below). The change in pol II enrichment is consistent with the change in GFP expression level.

We used the coefficient of variation (CV; standard deviation divided by the mean) to quantify the gene expression noise and plotted the noise versus mean for both GFP and mCherry in all our library strains (Fig 2A). We identified the strains with expression levels more than three standard deviation from the mean as “outliers”. For each outlier found in the initial screen, we confirmed its expression using three or more colonies and verified the integration site of the reporter with inverse PCR (Methods). As expected, there are more outliers in GFP expression than in mCherry. In total, 30 strains showed unusual expression in GFP but not in mCherry (“GFP outliers”), 1 in mCherry only, and 2 in both (S3 Table). The following investigation focused on the GFP outliers.

The GFP data in Fig 2A fall into four distinct regions (“profiles”). Most strains belong to profile 1 with close-to-average expression level and noise, while the rest can be divided into profile 2, 3, and 4 with “low expression / high noise”, “low expression / low noise”, and “high expression / low noise”, respectively. The profile 2–4 strains have close-to-average mCherry level (Fig 2A), showing that the unusual GFP expression is not due to global changes in the *MET3pr* activity. We selected one strain from each profile (strain 1–4 correspond to profile 1–4) and plotted their FACS data (Fig 2B). Note that the GFP expression in strain 2 and 3 are repressed to similar levels (0.59 vs 0.61), but the noise is significantly higher in strain 2 (p-value < 0.0001). This difference can be clearly visualized in fluorescent images of the two strains (Fig 2C). Since there is strong connection between noise and the underlying gene regulatory mechanism [26, 27], these data suggest that the *MET3pr* repression in profile 2 and 3 is caused by different mechanisms. Consistent with this idea, all of the profile 2 strains have *MET3pr*-GFP inserted in the silenced regions (HML, rDNA and telomere), and all of the profile 3 and 4 strains have the reporter in non-silencing “euchromatin” (Fig 2D). Eleven strains in profile 1 have the insertion sites in the sub-telomeric regions, which is consistent with a previous finding that some locations at the chromosome ends do not have the silencing effect [35].

GFP intensity reflects an integrated rate of transcription, translation, and post-transcriptional regulations. To test if the GFP outliers originate from altered transcriptional rates, we selected two strains from each profile and analyzed the RNA polymerase II (Pol II) distribution on the GFP and mCherry ORFs using chromatin immunoprecipitation (ChIP) (Methods). Comparing with profile 1, the pol II density over the GFP ORF is reduced in profile 2 and 3 (p-value < 0.0001 and 0.0027) and increased in profile 4 (p-value = 0.03), whereas it remains constant on the mCherry ORF (Fig 2E). Therefore, GFP expression in the outliers is modulated at the transcriptional level.

The transcriptional repression in profile 2 is likely due to Sir-dependent silencing that is known to spread over a few thousand bases [36]. Indeed, deletion of Sir2 leads to significant increase of the GFP expression in profile 2 strains (S3 Fig). We next investigated the regulatory mechanisms of profile 3 and 4.

### GFP repression in some profile 3 strains is due to transcriptional interference

**Fig 3:**
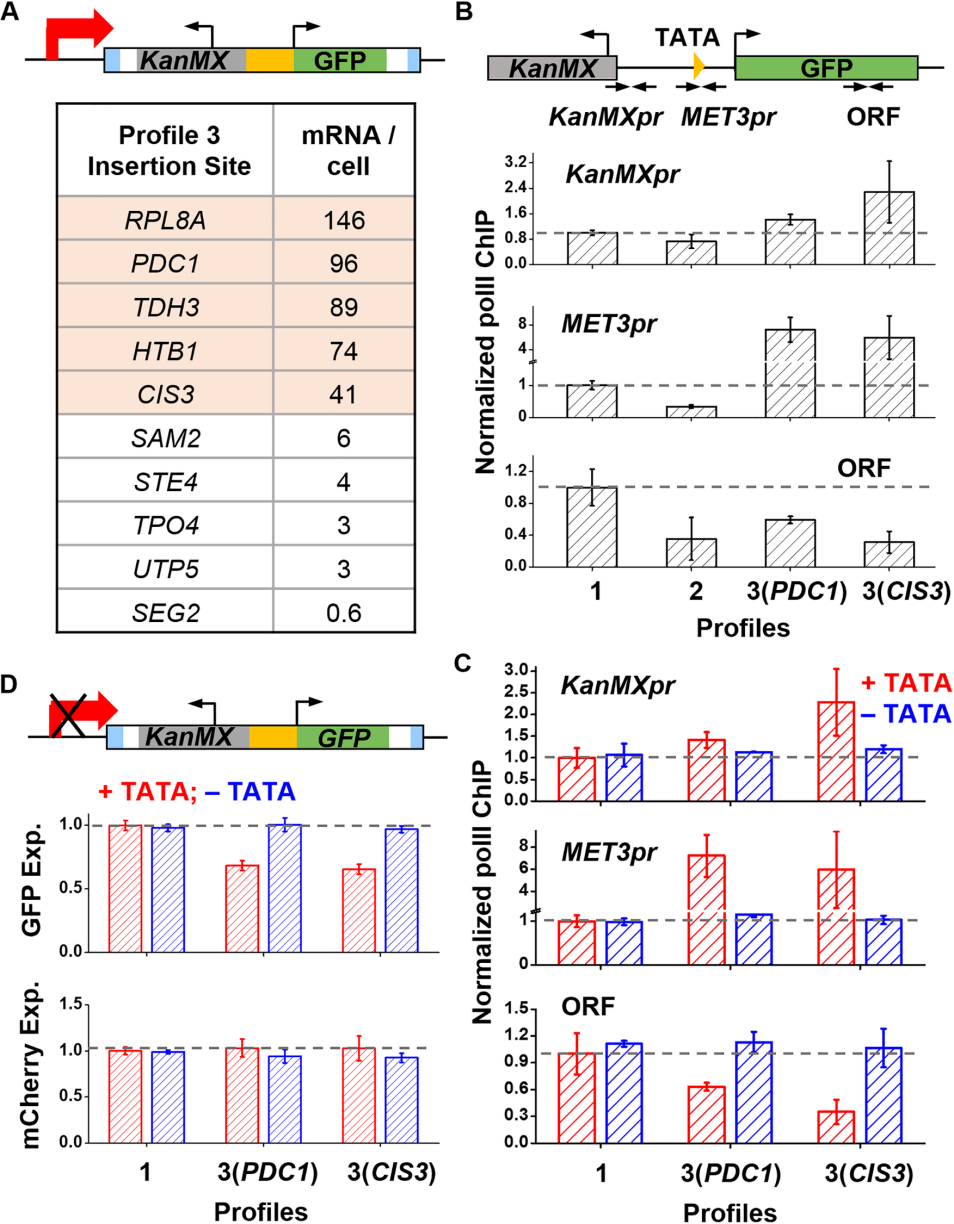
Repression in some profile 3 strains is due to transcriptional interference. **A)** Reporter insertion sites in profile 3 strains and the transcript levels of the local genes. Half of the profile 3 sites are located inside highly transcribed genes (highlighted in pink). **B)** Pol II ChIP over the *KanMX* promoter, *MET3pr*, and the GFP ORFs in four strains from profile 1, 2 and 3. The reporter cassette in the two profile 3 strains are located in *PDC1* and *CIS3*, two highly expressed genes. All ChIP signals were normalized by that in the profile 1 strains. The error bars represent the standard errors among three biological replicates. High pol II density over the promoters in profile 3 strains were detected. **C)** Effect of the upstream TATA deletion on pol II density. The reporter cassette are located in either *SNT2* (profile 1), or *PDC1* / *CIS3* (profile 3). Pol II ChIP signal was probed in identical locations as in (B). Deletion of the TATA box had no effect on pol II density over all three regions in the profile 1 strain (first two bars). In contrast, it restores the pol II density to the normal level in the two profile 3 strains. **D)** Effect of the upstream TATA deletion on GFP expression. Deletion of the *PDC1* or *CIS3* TATA box has no effect on mCherry expression, but restores the GFP expression to the same level as in the profile 1 strain.

We examined various properties of the reporter insertion sites in profile 3. We noticed that in these strains, the GFP reporters tend to be inserted into highly expressed ORFs (Fig 3A): according to the database in [37], 5 out of the 10 genes at the profile 3 insertion sites produce >40 mRNAs per cell, which is significantly higher in comparison to the rest of the genome (p-value < 2×10^−5^). This observation raised the possibility that the high-level transcription of the flanking genes may leak onto the *MET3pr* and interfere with its expression, as shown in some other cases [38, 39].

To test this idea, we selected two profile 3 strains containing *MET3pr*-GFP in highly expressed genes (*PDC1* and *CIS3*) and measured the pol II density over the GFP cassette. We probed the ChIP signals over the *KanMX* promoter, *MET3pr*, and GFP ORF (Fig 3B). We also included a profile 1 and a profile 2 strain as controls. Strains containing GFP but not mCherry were used here to maintain the *S*. *kud MET3pr* as a single copy. Consistent with Fig 2E, the profile 2 and 3 strains have less pol II over the GFP ORF. However, the two profile 3 strains have higher pol II density in the *KanMX* and *MET3* promoters (Fig 3B), supporting the idea that polymerases are “invading” from the upstream gene onto to the *MET3pr*. To test the causal relation between the invading transcription and GFP repression, we used CRISPR/Cas9 to delete the TATA elements of *PDC1* and *CIS3* and measured the resulting pol II density and GFP expression (Methods). Deletion of these TATA boxes restored pol II density and GFP expression to the profile 1 level (Fig 3C and 3D). These data show that GFP repression in these profile 3 strains are due to transcriptional interference.

There are also some profile 3 strains with GFP not in highly expressed genes (Fig 3A). We selected two of these strains (*SAM2* and *SEG2*) and performed pol II ChIP. Interestingly, the pol II density over the *MET3pr* is the same as in the profile 1 control, but the density over the GFP ORF is lower (S4 Fig). This result is different from the profile 2 strain where pol II density is lower in both regions (S4 Fig). These data indicate that the transcription of *MET3pr* in these profile 3 strains initiates at a normal level, but is curtailed in a subsequent step (e.g. transition from initiation to elongation or during elongation). The detailed mechanism of this repression is still unclear.

### GFP overexpression in profile 4 is due to clustering of co-regulated genes

**Fig 4:**
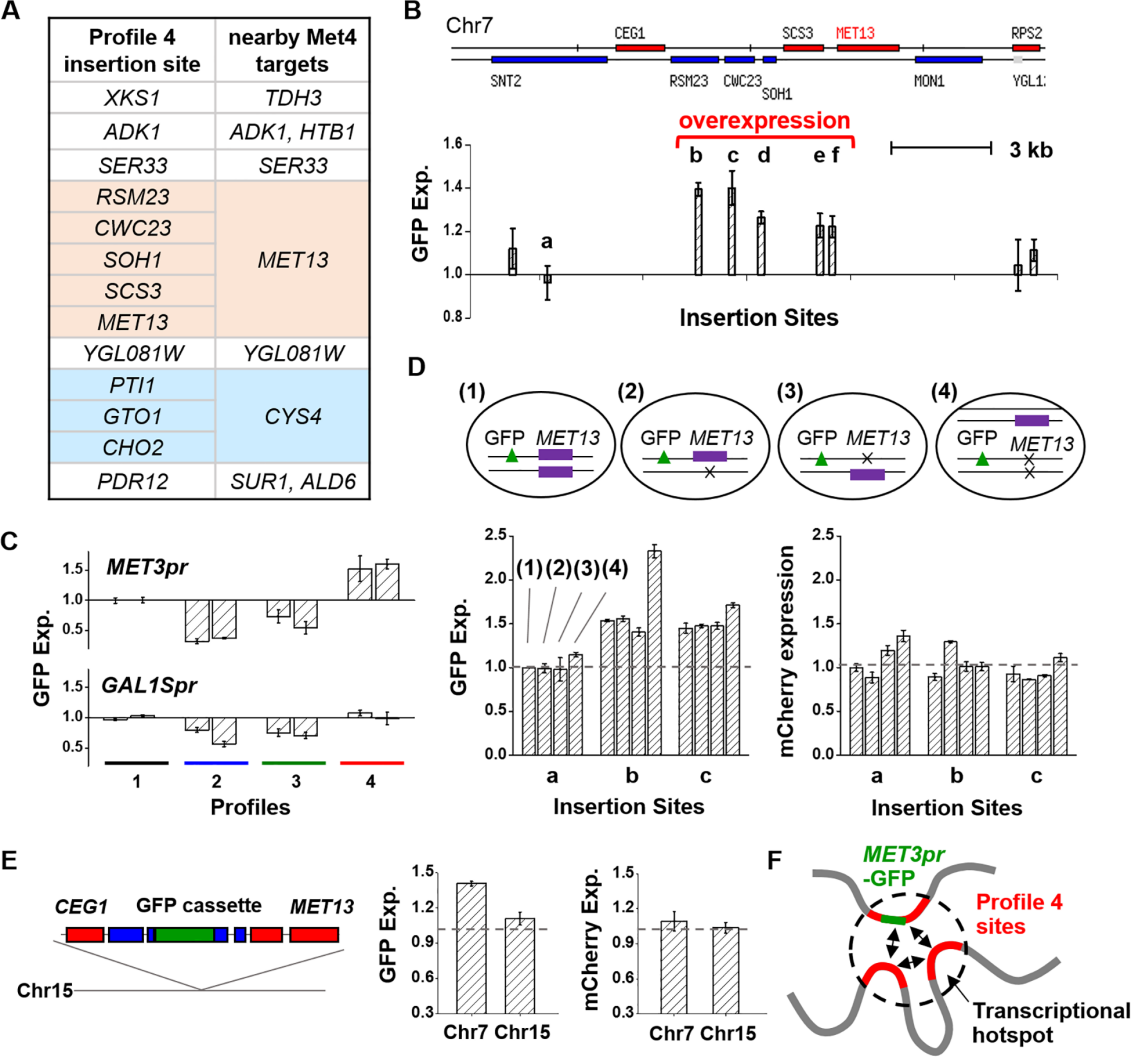
Overexpression in profile 4 is not due to local neighboring genes. **A)** Reporter insertion sites in profile 4 strains (left column). The insertion sites are either within or near Met4-targeted genes (right column). Some of these sites are close to each other in the genome (highlighted in colors). **B)** A group of the profile 4 insertion sites near the *MET13* gene on Chr7. *MET3pr-GFP* shows overexpression when inserted over a ~5kb region including multiple adjacent genes (from “b” to “f”). “a” is a nearby site profile 1 site. **C)** Promoter swapping test. We selected two strains from each profile and integrated the GFP reporter driven by the *GAL1Spr* instead of the *MET3pr*. *GAL1Spr-GFP* at profile 4 sites do not show overexpression, indicating that the overexpression is specific to the *MET3pr*. **D)** Deletion of the *MET13* allele does not affect the *MET3pr* overexpression. We started with three strains containing GFP reporter at site “a” (control), “b”, or “c” (1), and deleted the *trans* (2) or *cis* (3) copy of *MET13*, or both (4). The cluster of four bars in the lower plots correspond to the average GFP or mCherry expression with configurations (1)-(4). None of the *MET13* deletion eliminates the GFP overexpression at the “b” and “c” sites. **E)** GFP overexpression disappears when translocated with neighboring genes to a new genomic locus. The reporter cassette at the “c” site along with neighboring genes (from *CEG1* to *MET13*) was inserted into Chr15. The average GFP expression is reduced to the profile 1 level at this new location (p-value = 0.0038). **F)** Potential model for profile 4 overexpression. Some profile 4 sites may cluster at a transcriptional “hotspot”, allowing the nearby Met4-targeted genes to gain higher expression.

We next studied the mechanism of *MET3pr*-GFP overexpression in profile 4 strains. Unlike profile 3 strains, the reporter insertion sites in profile 4 strains involve genes with mild expressions (mRNAs per cell from 0.5 to 4) (S4 Table). Some profile 4 insertion sites are close to each other in the genome. For example, GFP reporters inserted into five consecutive genes, *RSM23*, *CWC23*, *SOH1*, *SCS3*, and *MET13*, all showed higher-than-average expression (p-values = 0.0004, 0.008, 0.0033, 0.0048, and 0.005 respectively; Fig 4A and 4B). This observation indicates that profile 4 overexpression is regulated by certain property of chromosomal regions, rather than that of individual genes. The profile 4 sites in Fig 4B are adjacent to *MET13*, which, similar to *MET3*, is also in the methionine metabolic pathway activated by transcription factor Met4 [40, 41]. It turns out that all profile 4 insertion sites are close to Met4-targeted genes (average distance 1.4 kb), which is highly significant comparing with random sites (p-value < 10^−4^; Fig 4A, S5 Fig; Methods). The reverse is not true: not all GFPs landed close to Met4-targeted genes are overexpressed.

Next, we investigated if the overexpression in profile 4 is specific to *MET3pr*. We carried out a “promoter swapping” experiment, in which we replaced *MET3pr* in the reporter cassette with *GAL1Spr* [42], an attenuated *GAL1* promoter with similar firing strength as the *MET3pr*. We integrated the new reporter into eight loci, two from each profile, induced the *GAL1Spr-*GFP expression with galactose, and measured the steady-state gene expression under the microscope. When inserted in the profile 2 and 3 sites, *GAL1Spr-*GFP continues to have lowered expression, as expected from the silencing and transcriptional interference mechanisms (Fig 4C). In contrast, *GAL1Spr-*GFP in profile 4 loci no longer shows overexpression (Fig 4C), indicating that the hyper-activity in profile 4 is *MET3pr*-specific.

A straightforward explanation for the data above is that some Met4-targeted genes (like *MET13*) may increase the local concentration of Met4 and/or other co-activators, and thus enhance the *MET3pr* activity in its vicinity. To test this idea, we deleted the entire *MET13* gene (including the promoter and the transcribed region) and measured the reporter expression. Since we conducted our experiments in diploids, we deleted the *MET13* either in *cis* or in *trans* relative to the reporter, or both (Methods). These deletions do not affect *MET3pr* activity globally because mCherry expression remains unchanged (Fig 4D). Importantly, the GFP expression is not reduced in any of the *MET13* deletion strains (Fig 4D), indicating that the overexpression is not due to the presence of a nearby *MET13* gene.

To test if the overexpression is due to the presence of other local genes, we took the reporter cassette in *CWC23* (site “c” in Fig 4B), together with the neighboring genomic sequences (3.8kb on one side and 5.5kb on the other), and inserted it into a profile 1 locus on Chr15 (Methods). This translocation reduces the GFP expression to the normal (profile 1) level without affecting the mCherry expression (Fig 4E). This result confirms that the GFP overexpression is not due to the neighboring *MET13* gene, nor any other genes within a few kb range.

**Fig 5:**
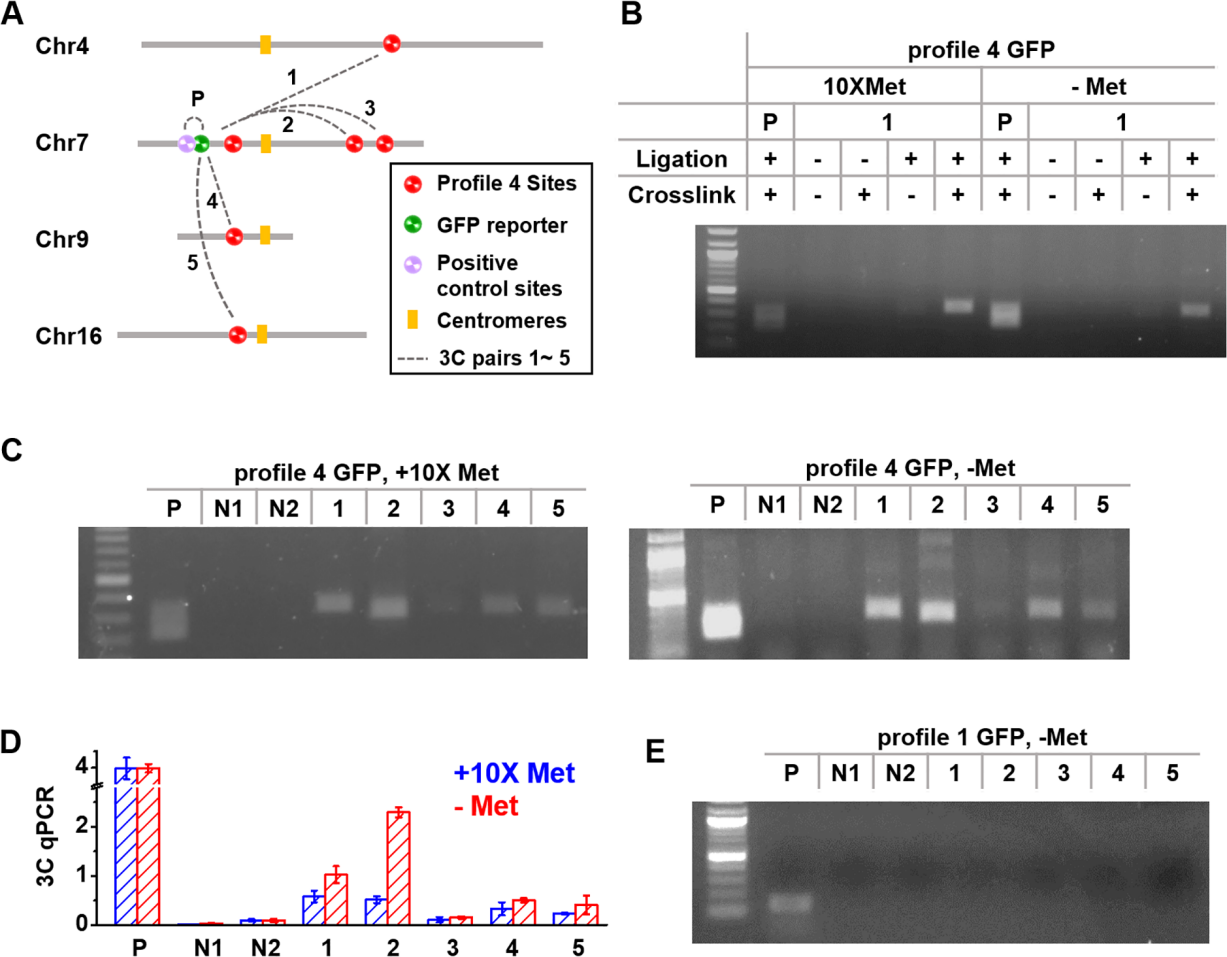
Long-distance interaction between profile 4 sites. **A)** 3C scheme to detect interactions between the GFP reporter (inserted into *CWC23*) and other profile 4 sites (1: *ADK1*, 2: *GTO1*, 3: *XKS1*, 4: *SER33*, 5: *PDR12*). **B)** 3C assay between the GFP reporter and *ADK1* (interaction #1) under both +/–methionine conditions in the presence of absence of crosslinking and/or ligation. P: positive control (3C between the GFP reporter and a *cis* region ~13kb away). The 3C product is only visible with ligation and crosslinking. **C)** 3C assay between the GFP reporter and five profile 4 sites (see A) under both +/–methionine conditions. Besides the same positive control (P), we also included two negative controls (N1 and N2; see text for detailed explanation). **D)** Quantification of the 3C signals in C using qPCR. The 3C signals of interaction 1, 2, 4, and 5 are increased after–Met induction (p-values are 0.0016, 0.0057, 0.049, and 0.0001), while that of the positive control remains constant. **E)** 3C assay between profile 4 sites 1–5 and the GFP reporter that is translocated to Chr15. The signals in 1–5 are no longer visible.

Based on the above evidence, we suspected that the overexpression is related to pathway-specific long-distance chromosomal organization. In particular, the profile 4 sites may be physically located in transcriptional “hotspot(s)” with high local concentration of Met4 and/or related factors that promote *MET3pr* firing (Fig 4F). If this model is correct, we may be able to detect interactions between at least some of the profile 4 sites. Therefore, we carried out 3C experiments to probe the interactions between a hyper-active *MET3pr-*GFP and other endogenous profile 4 loci (Fig 5A; Methods). Since previous reports indicate that chromosomal interactions in yeast may change with transcriptional status [19, 20, 43], the 3C assays were performed in either the presence or absence of methionine. We also performed the same measurement between *MET3pr-*GFP and a *cis* region ~13kb away as a positive control to ensure the success execution of the 3C assay (Methods).

As shown in Fig 5B, a 3C signal was detected between the *CWC23-*localized GFP and *ADK1*. As expected from the 3C procedure, this signal is absent without ligation and/or crosslinking (Fig 5B). This GFP reporter also makes contacts with most of the other profile 4 loci (Fig 5C). Although all of the 3C signals are visible in the presence or absence of methionine, the strength of the interactions increase by 1.5–4 fold in the activating condition (p-values = 0.0016, 0.0057, 0.049, 0.0001 for *ADK1*, *GTO1*, *SER33*, *PDR12*; Fig 5D). These results indicate that the overexpressed *MET3pr-*GFP is in physical proximity to many profile 4 sites before the induction, and they come closer after the induction.

To understand how specific the interactions occur at the profile 4 sites, we also included two negative controls in the 3C measurements (N1 and N2). N1 is a profile 1 site on Chr16 that has similar distance to the centromere as *ADK1*. Previous Hi-C experiments indicate that long-distance chromosomal interactions in yeast are partially determined by the Rabl configuration, in which sites with similar distance to the centromere tend to interact [11, 14]. We do not think the interactions seen in Fig 5C are based on this mechanism because the interacting loci have variable distances to centromeres (differing by >300kbp; S5 Table). Consistent with this idea, *ADK1* but not the N1 site shows interaction with GFP. N2 probes the interaction between the same *MET3pr-*GFP with *MET28*, a Met4-targeted gene that is a profile 1 site. No interaction was observed in this case (Fig 5C and 5E). It is important to point out that we have found profile 1 sites at other Met4-targeted genes that make contacts with the overexpressed *MET3pr-*GFP (S6A Fig). Interestingly, in comparison to Fig 5D, the strengths of these interactions show less changes in the activating condition (p-value < 0.005; S6B Fig), indicating that these sites do not cluster further with the profile 4 sites upon induction.

When we moved a profile 4 *MET3pr-*GFP together with 9.3kb of neighboring sequences to Chr15, the GFP no longer shows overexpression (Fig 4E). At this translocated site, *MET3pr-*GFP loses its interaction with all the profile 4 sites (Fig 5E), and the corresponding 3C signals were undetectable in qPCR. Overall, the correlation between GFP overexpression and its interaction with other profile 4 sites support the model in Fig 4F that profile 4 sites cluster to enhance *MET* gene expression.

### Endogenous genes at the profile 4 sites use long-distance interactions to enhance expression

**Fig 6:**
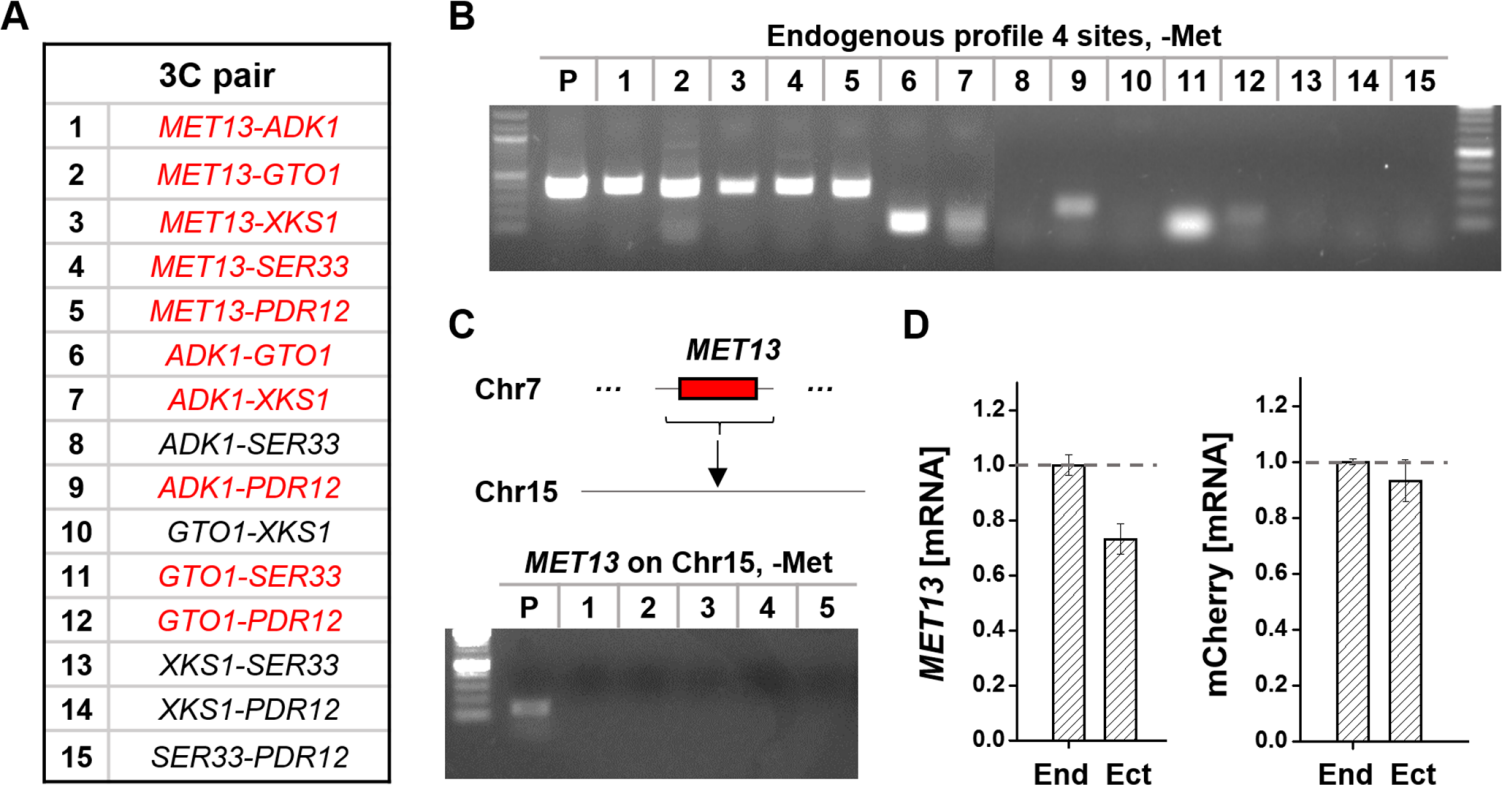
Long-distance interaction and gene regulation of the native profile 4 sites. **A)** A list of the 3C pairs between the native profile 4 sites. **B)** 3C assay between the gene pairs in A. The pairs with positive signals are labeled red in A. **C)** 3C assay between *MET13* and the rest of the profile 4 sites after translocation to Chr15. No interaction was detected at this new location. **D)** *MET13* expression measured by RT-PCR at the endogenous (End) or ectopic (Ect) location. The *MET13* mRNA level is significantly reduced on Chr15 (p-value < 0.001). The expression of the control (mCherry driven by *MET13pr* at *CDC20* locus) is unchanged.

In Figs 4 and 5, we focused on the chromosomal interaction and expression of GFP reporters. We suspect that the endogenous Met4-targeted genes at the profile 4 sites can also benefit from the same long-distance interactions to gain higher expression. To test this idea, we measured pair-wise interactions between the endogenous profile 4 sites (Fig 6A; Methods). Out of the 15 pairs, we detected 10 interactions (Fig 6B). In particular, *MET13* makes contacts with all the other profile 4 sites. We then moved the *MET13* gene from its endogenous location to a new location on Chr15 (same location as in Fig 4E; Methods). At this ectopic location, *MET13* loses its interactions with all of the other profile 4 sites (Fig 6C). Using RT-PCR to measure the average mRNA level of *MET13* before and after the translocation, we found that the *MET13* expression is significantly reduced at the new location (p-value = 0.0009; Fig 6D). The magnitude of the drop (~30%) is consistent with the difference in *MET3pr-*GFP expression at these two locations. In contrast, mCherry expression driven by the *MET13pr* at *CDC20* locus in these two strains are the same (Fig 6D). Therefore, similar to the *MET3pr-*GFP reporter, the endogenous *MET13* gene shows higher expression at its native genomic locus, which correlates with its long-distance interactions with other profile 4 loci.

## Discussion

### Three distinct mechanisms for gene regulation at a distance in yeast

Numerous intra- and inter-chromosomal interactions have been discovered in budding yeast, yet their role in gene regulation is far from clear. In this paper, we probed the effect of these interactions on gene expression by ectopically inserting an insulated reporter into thousands of genomic loci and characterizing the reporter activity at the single cell level. At most locations, the expression has similar average level and noise, indicating that the majority of long-distance chromosomal interactions detected by Hi-C do not play a significant role in gene regulation (at least for *MET3pr*). However, in a small fraction of locations, gene expression deviates from the norm and exhibits three distinct patterns including low expression / high noise, low expression / low noise, and high expression / low noise (profile 2, 3, and 4). Our follow-up studies indicate that profile 2 expression is due to the Sir2-mediated silencing, profile 3 is partially due to transcriptional interference, and profile 4 is due to 3D clustering of Met4-targeted genes. This assay may be used as a general platform to screen for functional long-distance chromosomal interactions that affect gene expression.

### Silencing and transcriptional interference generate different expression noise

Silencing and transcriptional interference are well-characterized mechanisms of gene repression. Interestingly, our data revealed that these two mechanisms can repress the average gene expression to a similar extent, but generate different levels of cell-to-cell variability. The detailed mechanism underlying this phenomenon requires further elucidation. Based on previous results [28, 30], we hypothesize that the two repression mechanisms have different time scales of action. Sir2-dependent silencing may be maintained at different levels from cell to cell for relatively long period of time (epigenetic memory), resulting in variable “silencing states”. In contrast, transcriptional interference is likely to occur multiple times during the *MET3pr* activation in each single cell, averaging out the stochasticity of this process and resulting in uniformly reduced transcription. For practical purposes, these two mechanisms can be used by synthetic biologists to engineer different gene expression noise with similar average level of expression.

### Overexpression and clustering

For the overexpression mechanism in the profile 4 strains, here is the evidence that we found: 1) overexpressed *MET3pr-*GFP tends to locate inside or close to a Met4-targeted gene, although the presence of this gene or other neighboring genes are not responsible for the overexpression (Fig 4A, 4D and 4E). 2) Overexpression is specific to the *MET3pr* (Fig 4C). 3) Overexpressed *MET3pr-*GFP contacts many other profile 4 loci, and the intensities of these interactions increase upon induction (Fig 5C and 5D). A large fraction of the profile 4 loci in the native genome also interact with each other (Fig 6B). 4) When either the overexpressed GFP reporter or the endogenous *MET13* gene is translocated to a different genomic locus, they lose the interactions with other profile 4 loci and show reduced expression (Figs 4E, 5E, 6C and 6D).

We interpret the interactions among the profile 4 sites as “clustering” of a subset of Met4-targeted genes. This interpretation is supported by previous findings that co-activated genes tend to cluster in 3D space [21, 22, 24, 44]. Interestingly, there seems to be a hierarchy among the profile 4 loci interactions: when we removed *MET13* from the cluster, not only the interactions between *MET13* and other profile 4 sites disappear, but also many interactions between *ADK1*/*XKS1*, *SER33*/*GTO1*, and *PDR12* (S7A Fig). In contrast, the interactions between *ADK1*-*XKS1* and *GTO1*-*SER33* remain present. These data indicate that *ADK1*-*XKS1* and *GTO1*-*SER33* may form “sub-clusters”, which are brought together by *MET13* (S7B Fig). Similar phenomenon has been observed in mammalian cells [23]. The translocation of *MET13* also results in mild but significant reduction in the expression of *ADK1*, *GTO1*, and *PDR12* (p-value < 0.034, 0.028, 0.022, respectively; S7C Fig). Overall, these data suggest that *MET13* is important for clustering and overexpression among profile 4 genes.

The detailed molecular mechanism underlying these clustering is not clear. Our data indicate that some degree of clustering occurs prior to induction, which is consistent with previous analysis showing that Met4-targets form significant 3D contacts even in rich media [22]. These interactions are either a passive consequence of chromosome folding, or actively mediated by some DNA-binding proteins or RNAs constitutively associated with these loci. The strengths of the interactions are quantitatively increased by transcriptional activation (Fig 5D), indicating that the transcription factors, transcription machinery, or RNAs may further enhance or stabilize the clustering.

In Figs 5 and 6, we show that clustering and overexpression are positively correlated. In particular, when translocated to a different genomic location, *MET3pr*-GFP and the endogenous *MET13* gene lose both the long-distance interaction and overexpression. Some Met4-targets show interaction with profile 4 sites but do not support *MET3pr*-GFP overexpression. The intensity of these interactions do not increase significantly after induction. Taken together, these results paint the following picture. A fraction of Met4-targeted genes are in physical proximity in the nucleus before induction, and a subset of these genes cluster more upon induction. Such clustering may directly lead to enhanced gene expression by creating a sub nuclear compartment with elevated local concentrations of transcription activators, GTFs, and/or pol II. A similar mechanism has been proposed to enhance transcription for other co-regulated gene clusters [23, 24, 45]. Future effort is needed to unambiguously dissect out the causal relation between clustering and overexpression.

## Materials and methods

### Plasmid and strain construction

Standard methods were used to construct the strains and plasmids. If not mentioned specifically, plasmids used in the study are derived from pRS yeast shuttle vectors. For the reporter cassette, the *S*. *Kud MET3pr* was flanked by an upstream *KanMX* gene that serves as a selective marker. The cassette contains *TEF1 (from Ashbya Gossypii)* and *ADH1* terminator at the 3’ end of the *KanMX* and GFP gene, respectively. Homologous sequence of the mTn transposon was added on each side of the cassette so that the reporter system can be integrated into the yeast insertion library (Open Biosystems) through homologous recombination. The library strain was derived from y800 diploid strain *(MATa leu2-D98cry1R/MATalpha leu2-D98CRY1 ade2-101 HIS3/ade2-101 his3-D200 ura3-52 caniR/ura3-52CAN1 lys2-801/lys2-801 CYH2/cyh2R trp1-1/TRP1 Cir0 carrying pGAL-cre (amp*,*ori*, *CEN*, *LEU2)*), with an mTn transposon sequence inserted as a single copy into thousands of different genomic loci [46]. The mCherry control was similarly constructed with the *URA3* marker. To avoid mCherry double integration, the integration site was chosen within an essential gene, *CDC20*, and only the strains with a single mCherry integration can survive. GFP and mCherry transformations were done consecutively in 96 plate format.

*MET13* deletion was carried out by replacing the entire *MET13* gene (from -237 to +2165 relative to the start of ORF) with *ADE2* gene. We chose this region starting from the end of transcription termination site of the upstream tandem gene *SCS3* to the termination site of the convergent downstream gene *MON1* [47]. Transformants were tested through tetrad dissection to determine which *MET13* allele was deleted. Strains with both alleles deleted were mated from two haploids both lacking *MET13*. Since *MET13* is essential for viability in–Met media, one copy of the *MET13* gene was integrated into a Chr15 locus (*LDS2*, 243695) in the double deletion strain (Figs 4D and 6C). To have a fair comparison of the 3C signal in Fig 6B and 6C, we deleted one *MET13* gene from the endogenous locus in Fig 6B, so that both strains contain single-copied *MET13*. The inserted *MET13* region in Fig 4E includes a ~9.3 kb fragment from the endogenous genome (ChrVII: 262709–274780) and the *MET3pr-GFP* reporter inserted in *CWC23*.

### FACS measurement and analysis

Strains containing the reporter genes were grown overnight in SCD + 10X Methionine (0.2 g/L) in a deep 96-well plate, spun down, washed, and diluted into SCD—Met media to OD_660_ ~ 0.05 for induction. After 6 h, samples were sonicated in Branson 5800 water bath for 20mins to break cell cluster into single cells and were then transferred into a shallow 96-well plate for flow cytometry measurement with BD LSR-Fortessa. The GFP is excited by the 488nm laser and filtered by a 525/50 PMT. The mCherry is excited by the 532nm laser and filtered by the 610/20 PMT. Data were quantified through Flowjo and Matlab program. We first gated the data based on the FSC (forward scattering) and SSC (side scattering) to select cells with regular size and shape, and gated these cells again based on the presence of both GFP and mCherry signal (a small fraction of cells, usually less than 2%, lose one fluorescence through loss of heterozygosity). We used these GFP-only and mCherry-only strains to calculate the crosstalk between the two fluorescence channels, and eliminated the crosstalk for the cells containing both GFP and mCherry. The final fluorescent signals were normalized based on the average expression of the profile 1 strains. “Outliers” are the strains with expression more than 3 standard deviation away from the mean.

### Inverse PCR

Protocol was adapted from a previously described method [48]. Cells were grown in 5 ml YEPD liquid media overnight to OD_660_ ~ 0.2, and genomic DNA was extracted through standard method. 5 ug of genomic DNA was used for AluI (4bp-cutter) digestion in a final volume of 50 ul overnight. 10 ul of digested DNA was added to 190 ul ligation mix containing 20 ul of 10x T4 DNA ligation Buffer, 0.2 μl of T4 DNA Ligase (NEB, 400U/μl) and 169.8 ul of water for intramolecular ligation at 16°C for > 4 h. The ligation products were ethanol precipitated and resuspended in 20 ul TE buffer. A pair of primers facing outwards in the GFP cassette were used to amplify the nearby unknown genomic sequences. PCR products were purified for Sanger sequencing. We confirmed the GFP reporter cassette insertion sites by mapping the sequencing results to the yeast genome.

### Fluorescence microscopy

We used the instrumentation and data acquisition platform as described in a previous study [49]. Cells were grown in SCD + 10X Met liquid media at 30°C to OD_660_ ~ 0.2, washed, and then transferred onto a SCD - Met agarose pad for induction. After 6 h, the agarose pad was put under the fluorescent microscope for imaging. The GFP and mCherry fluorescent intensity within each cell boundary were quantified. The crosstalk between GFP and mCherry fluorescence is negligible in this case.

### Pol II ChIP

ChIP protocol was modified from a previously described method [19]. Cells were grown in 100 ml SCD - Met to reach OD_660_ ~ 0.4 and then crosslinked by formaldehyde (final concentration 1%). After quenched with 6 ml of 2.5 M Glycine, these cells were harvested by centrifugation and disrupted by glass beads for 30 min at 4°C. The cell extract was then sonicated (Qsonica) to fragment chromatin to an average length of 350 bp. The whole cell extract was subjected to Rpb3 antibody (Biolegend) incubation followed by Protein A/G PLUS-Agarose (Santa Cruz Biotechnology, sc-2003) incubation. An aliquot of the whole cell extract was saved for input control. We extracted DNA from the input and immunoprecipitated samples and quantified them by qPCR analysis. See S6 Table for the primer sequences used in the qPCR.

### CRISPR/Cas9 gene editing of the TATA box

TATA consensus regions of two highly transcribed genes *PDC1* and *CIS3* were identified based on previous ChIP-exo study [50]. We used the one-vector CRISPR-Cas9 system [51] to delete these TATA elements. We inserted the 20mer guide DNA sequences (see S6 Table) into pML104, which also contains *TDH3pr*-driven Cas9 protein [51]. We transformed the modified pML104 plasmid into yeast together with a ~ 100 bp double stranded DNA fragment carrying the desired TATA-element deletion and a mutated PAM sequence (AGG to ACG). Transformants were selected on D-URA plates, confirmed with Sanger sequencing, and then transferred to D + FOA plates to pop-out the modified pML104 plasmid to avoid any potential side-effect of Cas9.

### Chromosome conformation capture (3C)

Protocols are adapted from Singh and Hampsey [8, 18]. Strains were incubated overnight at 30°C in SCD + 10 X Met and were then inoculated in a 50 ml SCD ± Met to an OD ~ 0.6–0.8. Cells were collected and resuspended in 10 ml of spheroplasting buffer (0.4 M sorbitol, 0.4 M KCl, 40 mM sodium phosphate buffer pH 7.2, and 0.5 mM MgCl_2_). 25 ul of Zymolyase 100T solution (20 mg/ml zymolyase 100-T, 2% glucose and 50 mM Tris-HCl, pH 7.5) were added at 30°C for 40 min to convert cells to spheroplasts. After washing twice in 10 ml of MES buffer (0.1 M MES, 1.2 M sorbitol, 1 mM EDTA pH8.0, and 0.5 M MgCl_2,_ adjust to pH 6.4), the spheroplasts were crosslinked by formaldehyde (final concentration 1%) for 15 min and quenched by 2.5 M glycine for 5 min. The crosslinked spheroplasts were washed twice and resuspended with 1X cutsmart Buffer (NEB) in 36.5 ul aliquots. Note that reactions should not be pooled as it will compromise the quality of the reaction. In one tube, we added 3.8 ul of 1% SDS (incubated for 10 min at 65°C), 4.4 ul 10% Triton X-100, and 60 U of HindIII to digest overnight with gentle rotation at 37°C. 8.6 ul of 10% SDS was added to inactivate HindIII by incubating at 65°C for 20 min. Digested chromatin was diluted in ligation mix to allow intramolecular ligation. For each tube, we added 74.5 ul of 10% Triton X-100, 74.5 ul of 10X ligation buffer, 8 ul of 10 mg/ml BSA, 8 ul of 100 mM ATP, 596 ul of ddH_2_O and 800 units of T4 DNA ligase, and incubated at 16°C for 4 hours. After the overnight treatment with proteinase K, we extracted the DNA with phenol/chloroform. Typically we get 5 ug of DNA at this step, and we use 100 ng for each 3C PCR amplification. See S6 Table for the primer sequences.

### Bioinformatic analysis of Met4-target enrichment near the profile 4 sites

We used a list of Met4 targets from Yeastract database for the bioinformatic analysis [52–55]. The list contains 405 documented Met4-activated genes from literature based on ChIP and microarray data. We calculated the average distance of all the profile 4 insertion sites to the closest Met4 targets (relative to the start of ORF). As a control, we selected 5000 random locations in the yeast genome and calculated their distances to the nearest Met4 target (see S5 Fig for histogram). The comparison between the two distances above show that profile 4 sites tend to locate near Met4-activated genes.

## Supporting information

S1 Fig Comparison between the homologous *MET3pr* in *S*.*cer* and *S*.*kud*. **A)** Activation dynamics of *S*.*kud MET3pr* (black) and *S*.*cer MET3pr* (red) measured by time-lapse fluorescent microscopy. Each trace represents *MET3pr*-GFP activity in a single cell during induction. The zero time point is the time of methionine removal. **B)** Steady state level of *S*.*kud MET3pr* and *S*.*cer MET3pr* integrated at three different locations: *ECM18* (profile 1), *YCL067C* (profile 2), and *TDH3* (profile 3). The data were normalized to the *ECM18* GFP intensity. Note that the two promoters show similar activation kinetics and steady state levels. **C)** PCR test of primer specificity. Primers complementary to the *S*.*kud MET3pr* sequence can amplify the *S*.*kud MET3pr* (right lane), but not the *S*.*cer MET3pr* (left lane).
(PPTX)

S2 Fig Distribution of the GFP reporter insertion sites. **A)** Locations of the insertion sites on the yeast chromosomes (grey bars). Some insertion sites fall into special regions, including telomeres (blue), mating loci (red), and centromeres (green). **B)** Categories of insertion sites based on their locations relative to the local genes.
(PPTX)

S3 Fig GFP repression in the profile 2 strains are due to Sir2-mediated silencing. We picked one profile 1 strain (control) and three profile 2 strains, where the GFP reporter is inserted into rDNA, telomere, and HML respectively, and carried out a heterozygous Sir2 deletion (Sir2 is known to be haploinsufficient). GFP expression is significantly increased in all the profile 2 strains (p-value < 0.01 in all three cases), but not in the control, confirming that GFP repression in profile 2 is indeed due to Sir2-mediated silencing.
(PPTX)

S4 Fig Pol II ChIP over the reporter in four strains from profile 1, 2 and 3. The reporter cassette in the two profile 3 strains are located in *SAM2* and *SEG2*, two moderately expressed genes. 3(1): *SAM2*; 3(2): *SEG2*. All ChIP signals were normalized by that in the profile 1 strain. The error bars represent the standard errors among three biological replicates. Different from Fig 3B, the profile 3 strains here have normal pol II density over the *KanMXpr* and *MET3pr* but lower density over the GFP ORF, indicating that they are repressed by a different mechanism.
(PPTX)

S5 Fig Histogram of the distances to the nearest Met4-targeted genes from randomly selected locations. We obtained 405 documented Met4-targeted genes from literature based on ChIP and microarray data [1–4]. We generated 5000 random locations in the yeast genome and calculated their distances to the nearest Met4 targets (start of the ORF). The plot above is the histogram of the distances. Comparing with these random locations, profile 4 insertion sites are significantly closer to Met4-targets (p-value < 1 X 10^−4^).
(PPTX)

S6 Fig Interactions between *MET3pr* and profile 1 Met4-targetd sites and their quantitative changes upon methionine induction. **A)** Interactions between *MET3pr* and selected profile 1 Met4-targetd sites. **B)** 3C signal change of profile 4 and profile 1 sites (2–9 in A) before and after induction. Signals are normalized by the positive control (P).The dash line represents the average 3C signal increase of profile 4 interactions. Overall, profile 1 sites have less changes in interaction strengths comparing with profile 4 sites (p-value = 0.0022).
(PPTX)

S7 Fig Interactions between native profile 4 sites before and after *MET13* translocation. **A)** 3C assay measuring all the pair-wise interactions listed in Fig 6A. **B)** Model of interactions between profile 4 sites before and after *MET13* translocation. The interactions between *ADK1*/*XKS1*, *SER33*/*GTO1*, and *PDR12* disappear after *MET13* translocation, indicating that these interactions may be bridged by *MET13*. **C)** mRNA levels of the profile 4 genes measured by RT-PCR (normalized by that of *ACT1*). The expression of *MET13*, *ADK1*, *GTO1*, and *PDR12* have significant decrease. The p-values are 0.0007, 0.034, 0.028, and 0.022, respectively.
(PPTX)

S1 Table Strain list. (PPTX)

S2 Table Library strain list and expression data. (XLSX)

S3 Table Outlier strains and expression data. (PPTX)

S4 Table mRNA level of the endogenous genes at profile 4 insertion sites. (PPTX)

S5 Table Distance between the sites tested in 3C to centromeres. (PPTX)

S6 Table Primer list. (PPTX)
