# Supplementary material for: Three distinct mechanisms of long-distance modulation of gene expression in yeast
Source: PLoS Genet. 2017 Apr 20;13(4):e1006736. doi: 10.1371/journal.pgen.1006736 (PMC5417705; doi:10.1371/journal.pgen.1006736)
Supplement: S1 Fig — A) Activation dynamics of S.kud MET3pr (black) and S.cer MET3pr (red) measured by time-lapse fluorescent microscopy. Each trace represents MET3pr-GFP activity in a single cell during induction. The zero time point is the time of methionine removal. B) Steady state level of S.kud MET3pr and S.cer MET3pr integrated at three different locations: ECM18 (profile 1), YCL067C (profile 2), and TDH3 (profile 3). The data were normalized to the ECM18 GFP intensity. Note that the two promoters show similar activation kinetics and steady state levels. C) PCR test of primer specificity. Primers complementary to the S.kud MET3pr sequence can amplify the S.kud MET3pr (right lane), but not the S.cer MET3pr (left lane). (PPTX) [file pgen.1006736.s001.pptx]

## Slide 1
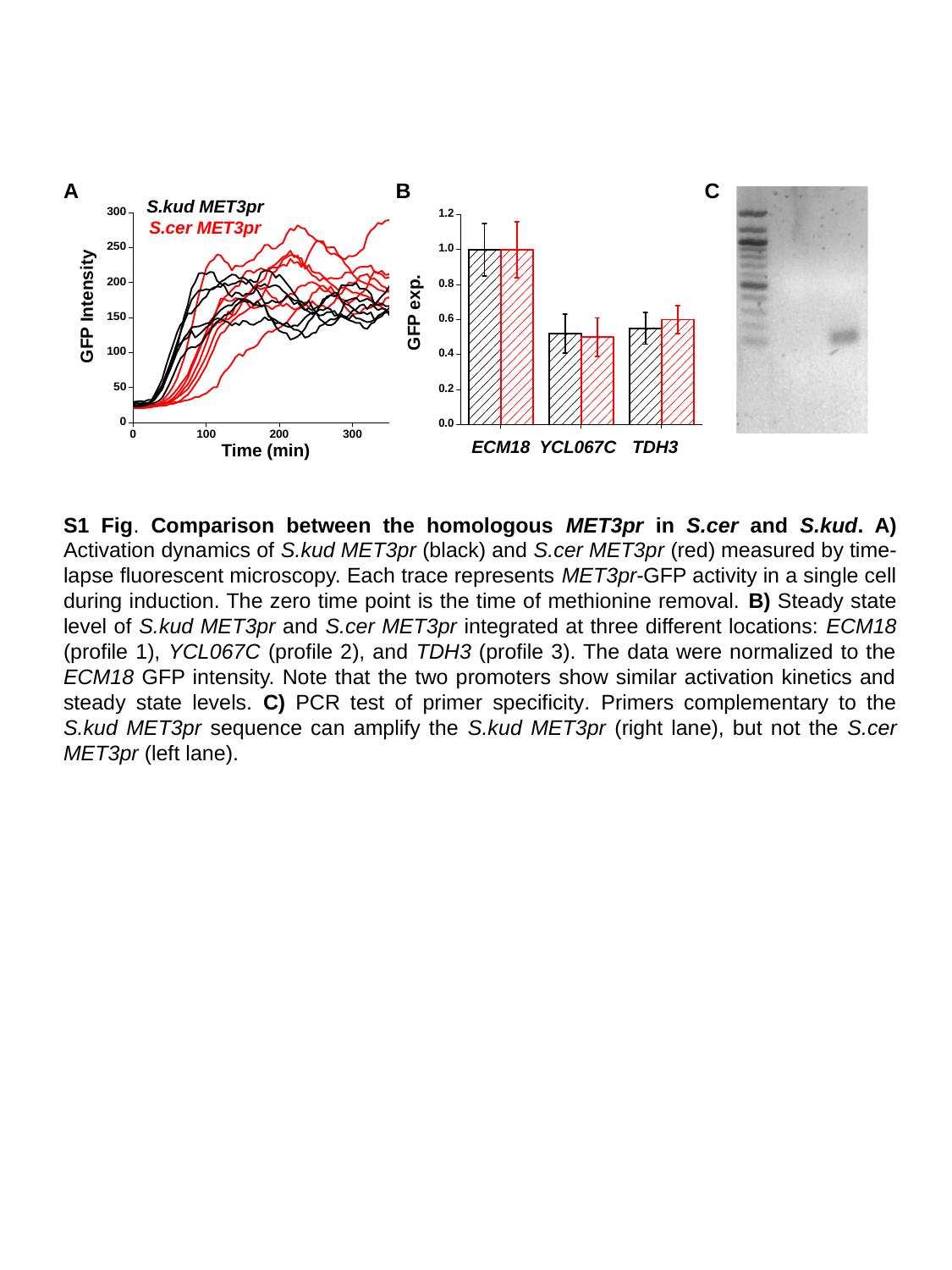

A
B
C
S.kud MET3pr
S.cer MET3pr
GFP Intensity
GFP exp.
ECM18
YCL067C
TDH3
Time (min)
S1 Fig. Comparison between the homologous MET3pr in S.cer and S.kud. A) Activation dynamics of S.kud MET3pr (black) and S.cer MET3pr (red) measured by time-lapse fluorescent microscopy. Each trace represents MET3pr-GFP activity in a single cell during induction. The zero time point is the time of methionine removal. B) Steady state level of S.kud MET3pr and S.cer MET3pr integrated at three different locations: ECM18 (profile 1), YCL067C (profile 2), and TDH3 (profile 3). The data were normalized to the ECM18 GFP intensity. Note that the two promoters show similar activation kinetics and steady state levels. C) PCR test of primer specificity. Primers complementary to the S.kud MET3pr sequence can amplify the S.kud MET3pr (right lane), but not the S.cer MET3pr (left lane).
