# Supplementary material for: Three distinct mechanisms of long-distance modulation of gene expression in yeast
Source: PLoS Genet. 2017 Apr 20;13(4):e1006736. doi: 10.1371/journal.pgen.1006736 (PMC5417705; doi:10.1371/journal.pgen.1006736)
Supplement: S2 Fig — A) Locations of the insertion sites on the yeast chromosomes (grey bars). Some insertion sites fall into special regions, including telomeres (blue), mating loci (red), and centromeres (green). B) Categories of insertion sites based on their locations relative to the local genes. (PPTX) [file pgen.1006736.s002.pptx]

## Slide 1
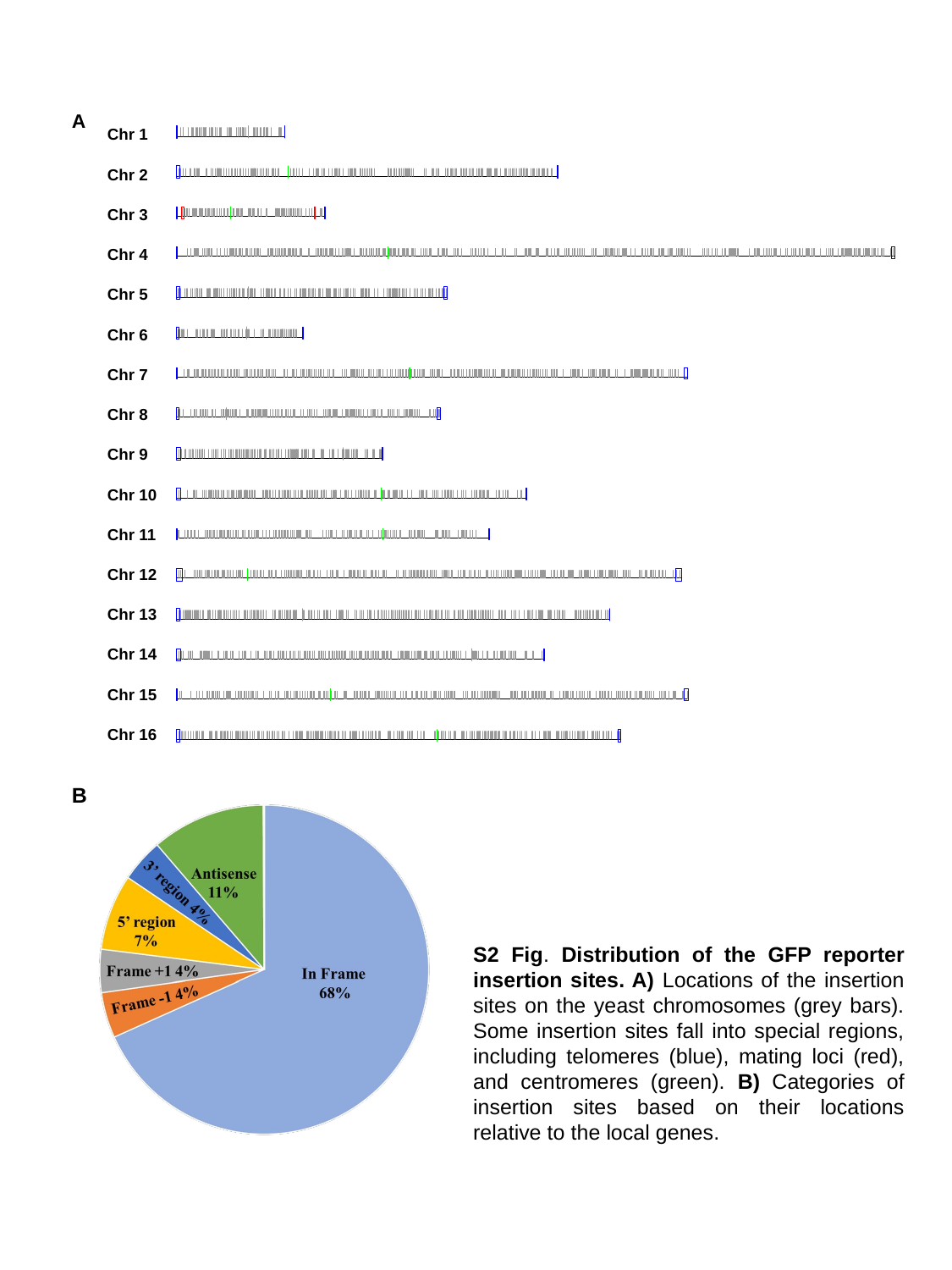

A
Chr 1
Chr 2
Chr 3
Chr 4
Chr 5
Chr 6
Chr 7
Chr 8
Chr 9
Chr 10
Chr 11
Chr 12
Chr 13
Chr 14
Chr 15
Chr 16
B
S2 Fig. Distribution of the GFP reporter insertion sites. A) Locations of the insertion sites on the yeast chromosomes (grey bars). Some insertion sites fall into special regions, including telomeres (blue), mating loci (red), and centromeres (green). B) Categories of insertion sites based on their locations relative to the local genes.
