# Supplementary material for: Three distinct mechanisms of long-distance modulation of gene expression in yeast
Source: PLoS Genet. 2017 Apr 20;13(4):e1006736. doi: 10.1371/journal.pgen.1006736 (PMC5417705; doi:10.1371/journal.pgen.1006736)
Supplement: S3 Fig — We picked one profile 1 strain (control) and three profile 2 strains, where the GFP reporter is inserted into rDNA, telomere, and HML respectively, and carried out a heterozygous Sir2 deletion (Sir2 is known to be haploinsufficient). GFP expression is significantly increased in all the profile 2 strains (p-value < 0.01 in all three cases), but not in the control, confirming that GFP repression in profile 2 is indeed due to Sir2-mediated silencing. (PPTX) [file pgen.1006736.s003.pptx]

## Slide 1
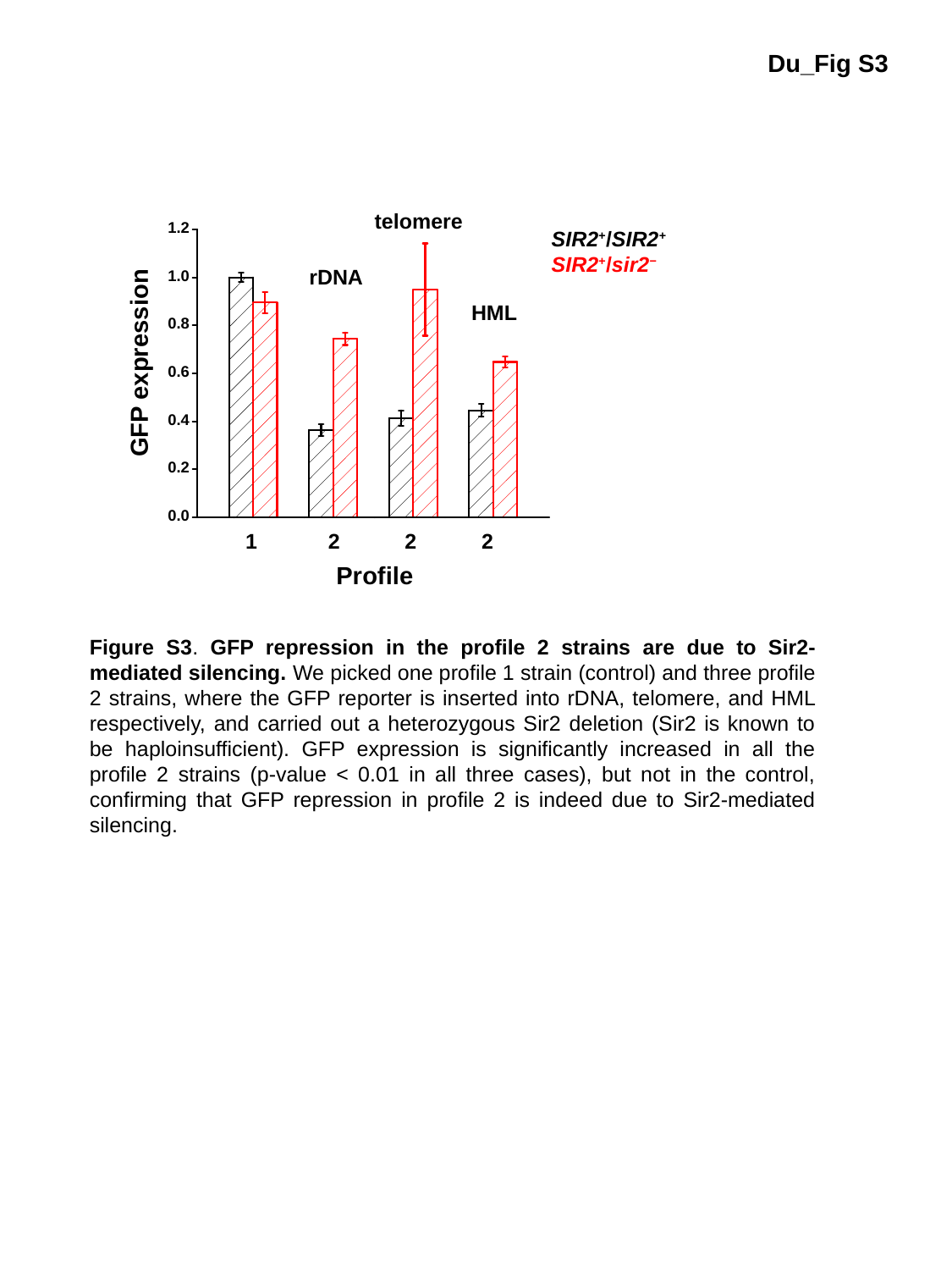

Du_Fig S3
 telomere
SIR2+/SIR2+
SIR2+/sir2−
 rDNA
 HML
GFP expression
1 2 2 2
 Profile
Figure S3. GFP repression in the profile 2 strains are due to Sir2-mediated silencing. We picked one profile 1 strain (control) and three profile 2 strains, where the GFP reporter is inserted into rDNA, telomere, and HML respectively, and carried out a heterozygous Sir2 deletion (Sir2 is known to be haploinsufficient). GFP expression is significantly increased in all the profile 2 strains (p-value < 0.01 in all three cases), but not in the control, confirming that GFP repression in profile 2 is indeed due to Sir2-mediated silencing.
