# Supplementary material for: Three distinct mechanisms of long-distance modulation of gene expression in yeast
Source: PLoS Genet. 2017 Apr 20;13(4):e1006736. doi: 10.1371/journal.pgen.1006736 (PMC5417705; doi:10.1371/journal.pgen.1006736)
Supplement: S4 Fig — The reporter cassette in the two profile 3 strains are located in SAM2 and SEG2, two moderately expressed genes. 3(1): SAM2; 3(2): SEG2. All ChIP signals were normalized by that in the profile 1 strain. The error bars represent the standard errors among three biological replicates. Different from Fig 3B, the profile 3 strains here have normal pol II density over the KanMXpr and MET3pr but lower density over the GFP ORF, indicating that they are repressed by a different mechanism. (PPTX) [file pgen.1006736.s004.pptx]

## Slide 1
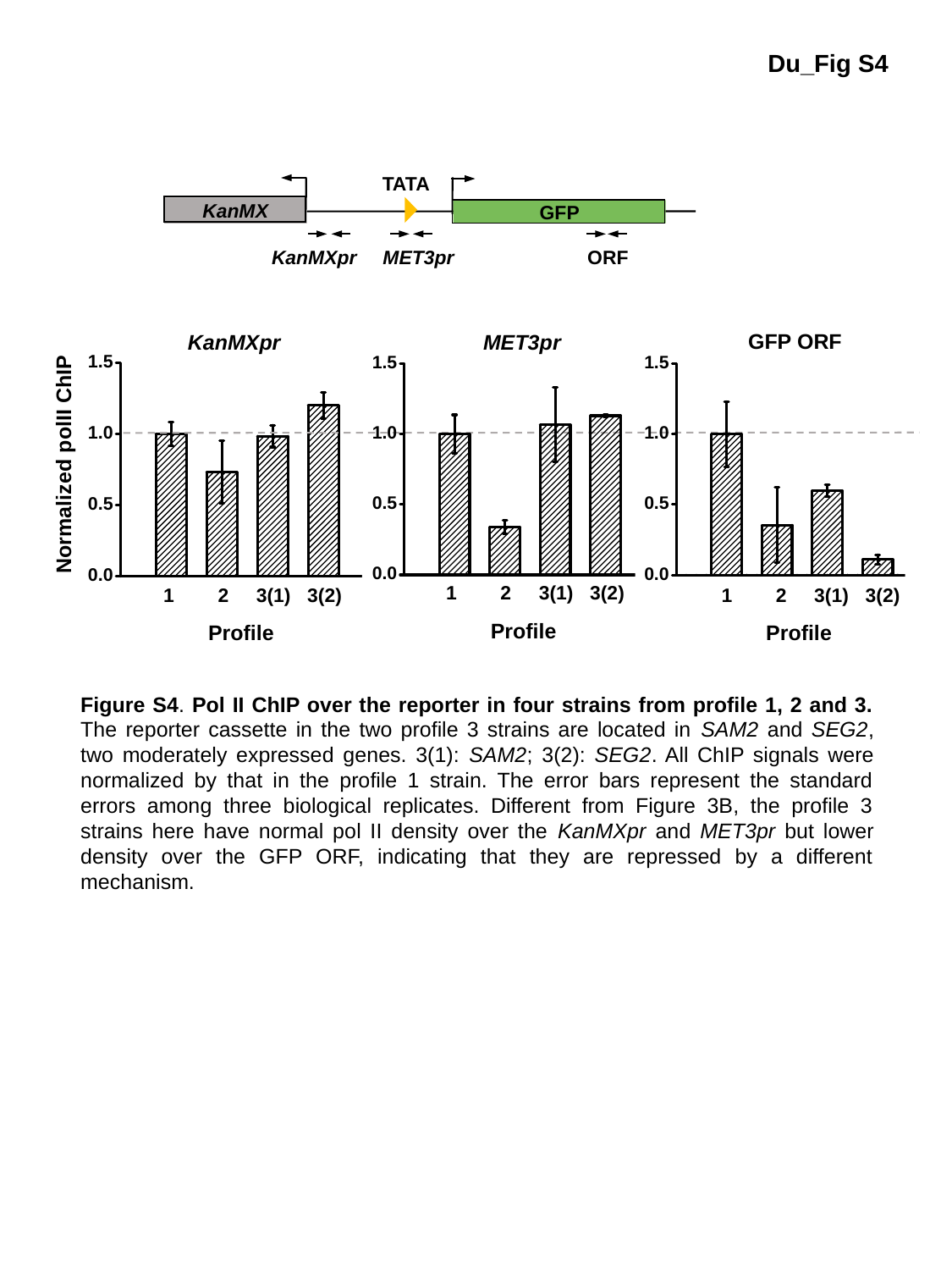

Du_Fig S4
TATA
GFP
KanMX
KanMXpr
MET3pr
ORF
GFP ORF
MET3pr
KanMXpr
Normalized polII ChIP
 1 2 3(1) 3(2)
 1 2 3(1) 3(2)
 1 2 3(1) 3(2)
 Profile
 Profile
 Profile
Figure S4. Pol II ChIP over the reporter in four strains from profile 1, 2 and 3. The reporter cassette in the two profile 3 strains are located in SAM2 and SEG2, two moderately expressed genes. 3(1): SAM2; 3(2): SEG2. All ChIP signals were normalized by that in the profile 1 strain. The error bars represent the standard errors among three biological replicates. Different from Figure 3B, the profile 3 strains here have normal pol II density over the KanMXpr and MET3pr but lower density over the GFP ORF, indicating that they are repressed by a different mechanism.
