# Supplementary material for: Three distinct mechanisms of long-distance modulation of gene expression in yeast
Source: PLoS Genet. 2017 Apr 20;13(4):e1006736. doi: 10.1371/journal.pgen.1006736 (PMC5417705; doi:10.1371/journal.pgen.1006736)
Supplement: S5 Fig — We obtained 405 documented Met4-targeted genes from literature based on ChIP and microarray data [1–4]. We generated 5000 random locations in the yeast genome and calculated their distances to the nearest Met4 targets (start of the ORF). The plot above is the histogram of the distances. Comparing with these random locations, profile 4 insertion sites are significantly closer to Met4-targets (p-value < 1 X 10−4). (PPTX) [file pgen.1006736.s005.pptx]

## Slide 1
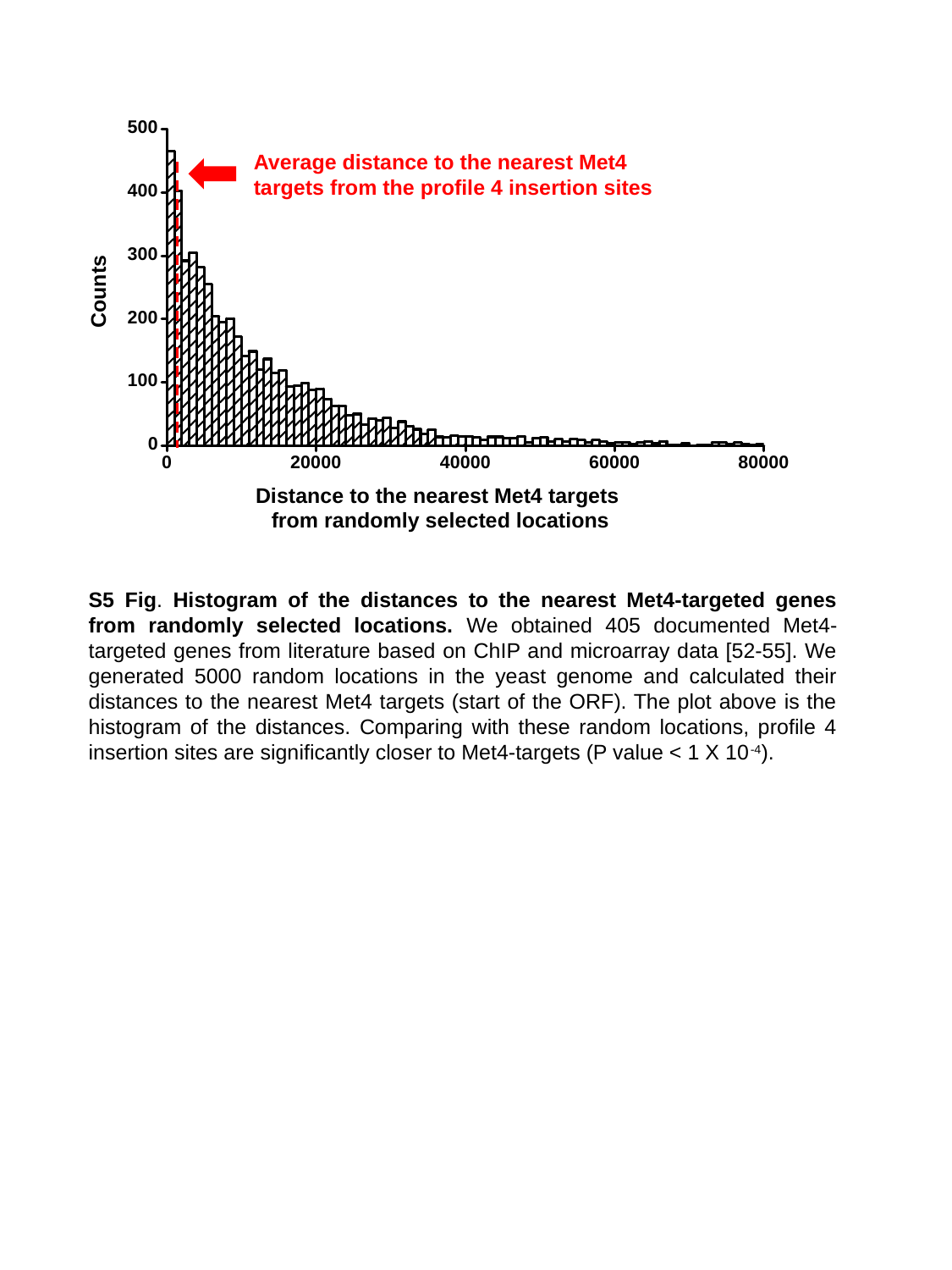

Average distance to the nearest Met4 targets from the profile 4 insertion sites
Counts
# Distance to the nearest Met4 targets from randomly selected locations
S5 Fig. Histogram of the distances to the nearest Met4-targeted genes from randomly selected locations. We obtained 405 documented Met4-targeted genes from literature based on ChIP and microarray data [52-55]. We generated 5000 random locations in the yeast genome and calculated their distances to the nearest Met4 targets (start of the ORF). The plot above is the histogram of the distances. Comparing with these random locations, profile 4 insertion sites are significantly closer to Met4-targets (P value < 1 X 10-4).
