# Supplementary material for: Three distinct mechanisms of long-distance modulation of gene expression in yeast
Source: PLoS Genet. 2017 Apr 20;13(4):e1006736. doi: 10.1371/journal.pgen.1006736 (PMC5417705; doi:10.1371/journal.pgen.1006736)
Supplement: S6 Fig — A) Interactions between MET3pr and selected profile 1 Met4-targetd sites. B) 3C signal change of profile 4 and profile 1 sites (2–9 in A) before and after induction. Signals are normalized by the positive control (P).The dash line represents the average 3C signal increase of profile 4 interactions. Overall, profile 1 sites have less changes in interaction strengths comparing with profile 4 sites (p-value = 0.0022). (PPTX) [file pgen.1006736.s006.pptx]

## Slide 1
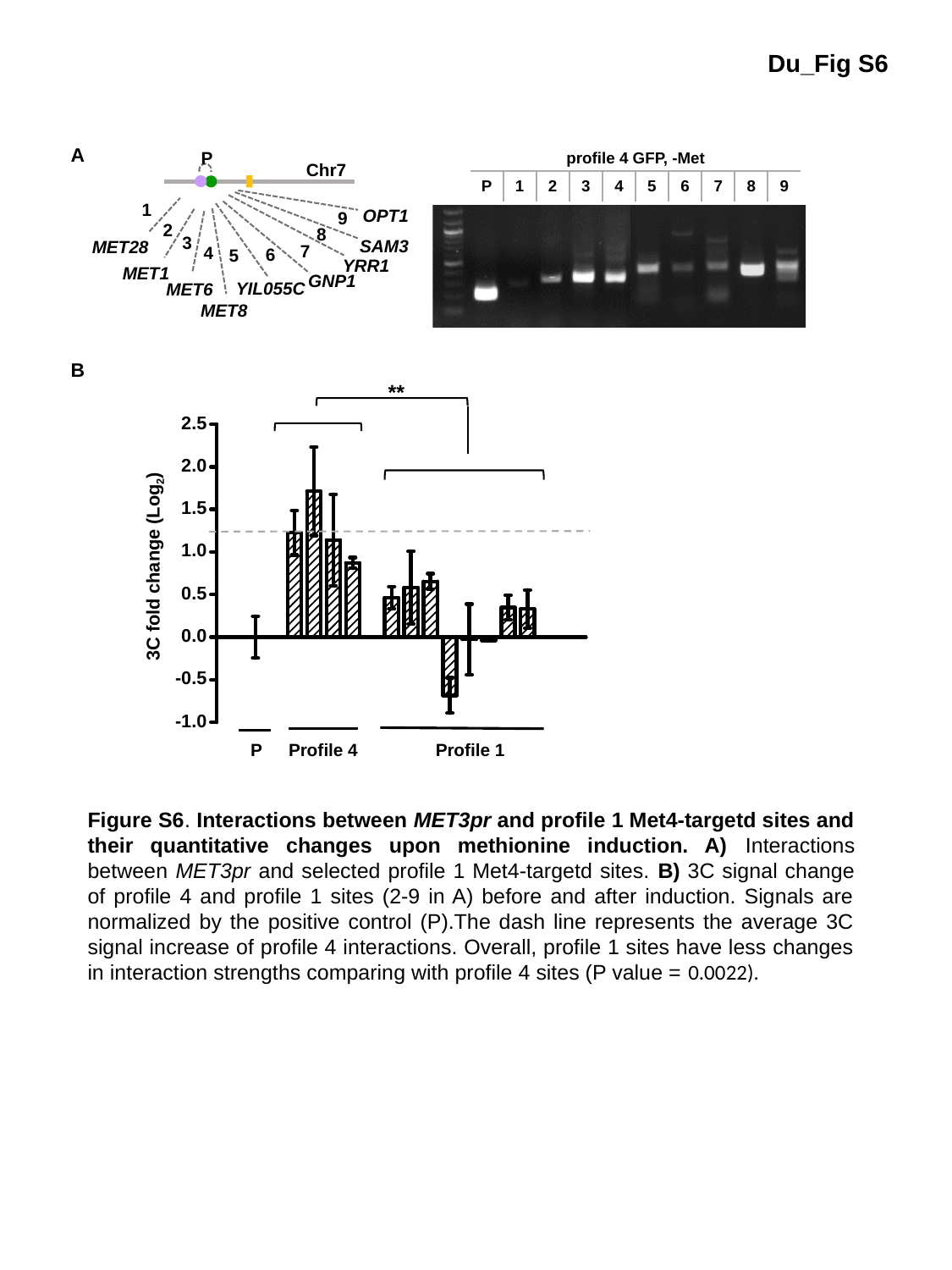

Du_Fig S6
A
P
| profile 4 GFP, -Met | | | | | | | | | |
| --- | --- | --- | --- | --- | --- | --- | --- | --- | --- |
| P | 1 | 2 | 3 | 4 | 5 | 6 | 7 | 8 | 9 |
Chr7
1
OPT1
9
2
8
3
SAM3
MET28
7
4
6
5
YRR1
MET1
GNP1
YIL055C
MET6
MET8
B
**
3C fold change (Log2)
P
Profile 4
Profile 1
Figure S6. Interactions between MET3pr and profile 1 Met4-targetd sites and their quantitative changes upon methionine induction. A) Interactions between MET3pr and selected profile 1 Met4-targetd sites. B) 3C signal change of profile 4 and profile 1 sites (2-9 in A) before and after induction. Signals are normalized by the positive control (P).The dash line represents the average 3C signal increase of profile 4 interactions. Overall, profile 1 sites have less changes in interaction strengths comparing with profile 4 sites (P value = 0.0022).
