# Supplementary material for: Three distinct mechanisms of long-distance modulation of gene expression in yeast
Source: PLoS Genet. 2017 Apr 20;13(4):e1006736. doi: 10.1371/journal.pgen.1006736 (PMC5417705; doi:10.1371/journal.pgen.1006736)
Supplement: S7 Fig — A) 3C assay measuring all the pair-wise interactions listed in Fig 6A. B) Model of interactions between profile 4 sites before and after MET13 translocation. The interactions between ADK1/XKS1, SER33/GTO1, and PDR12 disappear after MET13 translocation, indicating that these interactions may be bridged by MET13. C) mRNA levels of the profile 4 genes measured by RT-PCR (normalized by that of ACT1). The expression of MET13, ADK1, GTO1, and PDR12 have significant decrease. The p-values are 0.0007, 0.034, 0.028, and 0.022, respectively. (PPTX) [file pgen.1006736.s007.pptx]

## Slide 1
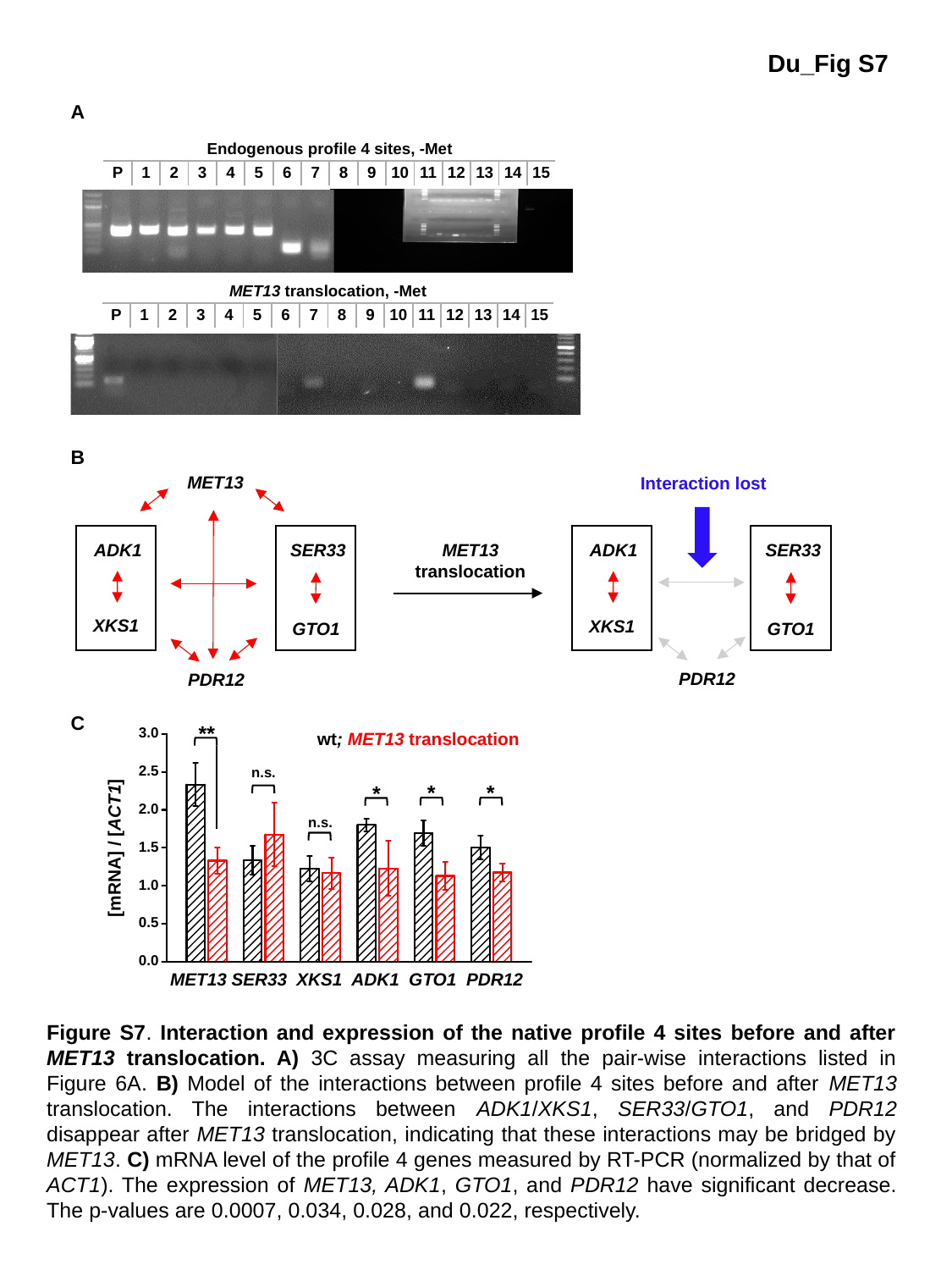

Du_Fig S7
A
| Endogenous profile 4 sites, -Met | | | | | | | | | | | | | | | |
| --- | --- | --- | --- | --- | --- | --- | --- | --- | --- | --- | --- | --- | --- | --- | --- |
| P | 1 | 2 | 3 | 4 | 5 | 6 | 7 | 8 | 9 | 10 | 11 | 12 | 13 | 14 | 15 |
| MET13 translocation, -Met | | | | | | | | | | | | | | | |
| --- | --- | --- | --- | --- | --- | --- | --- | --- | --- | --- | --- | --- | --- | --- | --- |
| P | 1 | 2 | 3 | 4 | 5 | 6 | 7 | 8 | 9 | 10 | 11 | 12 | 13 | 14 | 15 |
B
MET13
Interaction lost
MET13 translocation
ADK1
SER33
SER33
ADK1
XKS1
XKS1
GTO1
GTO1
PDR12
PDR12
C
**
wt; MET13 translocation
n.s.
*
*
*
n.s.
[mRNA] / [ACT1]
MET13 SER33 XKS1 ADK1 GTO1 PDR12
Figure S7. Interaction and expression of the native profile 4 sites before and after MET13 translocation. A) 3C assay measuring all the pair-wise interactions listed in Figure 6A. B) Model of the interactions between profile 4 sites before and after MET13 translocation. The interactions between ADK1/XKS1, SER33/GTO1, and PDR12 disappear after MET13 translocation, indicating that these interactions may be bridged by MET13. C) mRNA level of the profile 4 genes measured by RT-PCR (normalized by that of ACT1). The expression of MET13, ADK1, GTO1, and PDR12 have significant decrease. The p-values are 0.0007, 0.034, 0.028, and 0.022, respectively.
