# Supplementary material for: Three distinct mechanisms of long-distance modulation of gene expression in yeast
Source: PLoS Genet. 2017 Apr 20;13(4):e1006736. doi: 10.1371/journal.pgen.1006736 (PMC5417705; doi:10.1371/journal.pgen.1006736)
Supplement: S1 Table — (PPTX) [file pgen.1006736.s008.pptx]

## Slide 1
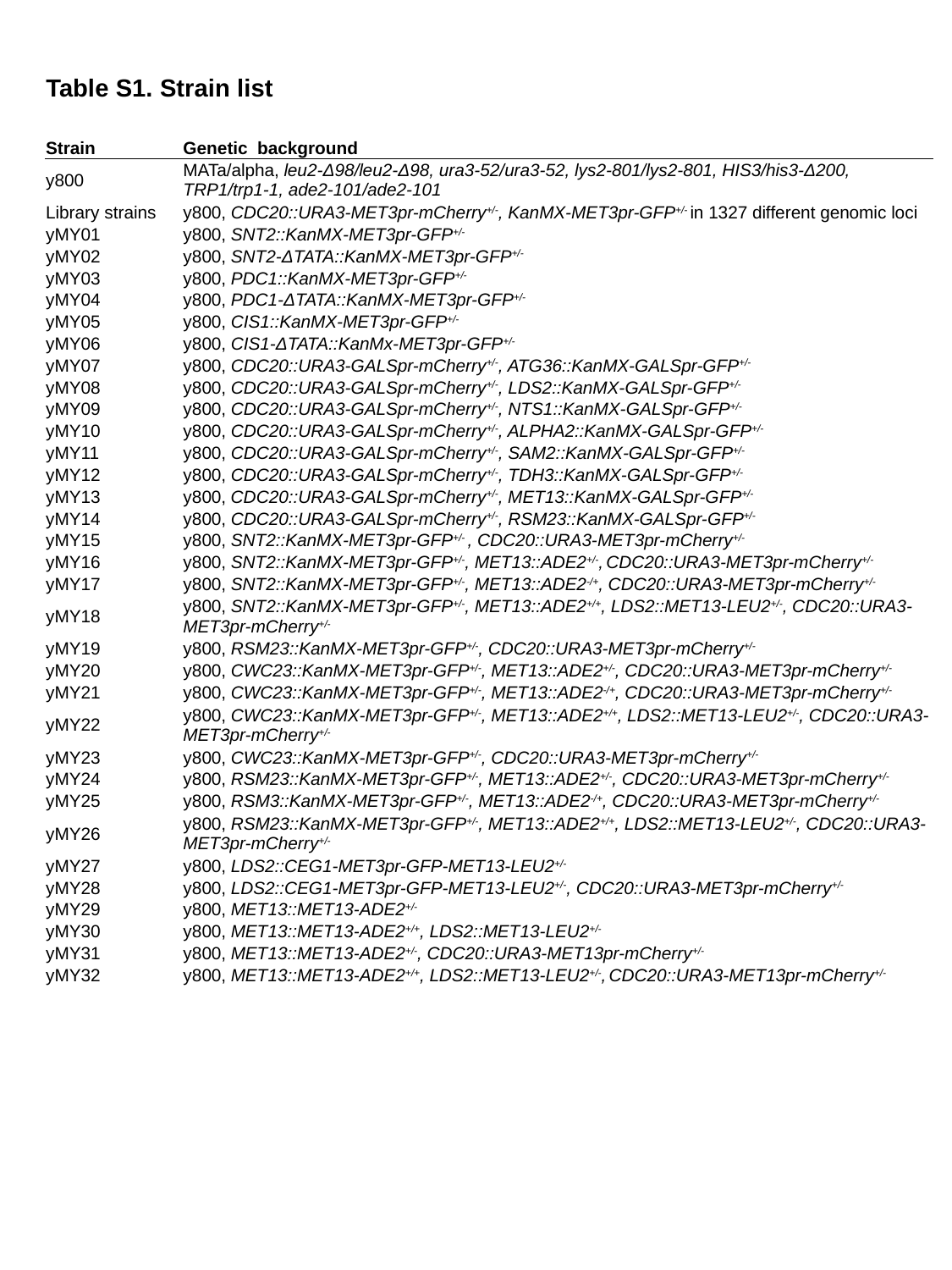

Table S1. Strain list
| Strain | Genetic background |
| --- | --- |
| y800 | MATa/alpha, leu2-Δ98/leu2-Δ98, ura3-52/ura3-52, lys2-801/lys2-801, HIS3/his3-Δ200, TRP1/trp1-1, ade2-101/ade2-101 |
| Library strains | y800, CDC20::URA3-MET3pr-mCherry+/-, KanMX-MET3pr-GFP+/- in 1327 different genomic loci |
| yMY01 | y800, SNT2::KanMX-MET3pr-GFP+/- |
| yMY02 | y800, SNT2-ΔTATA::KanMX-MET3pr-GFP+/- |
| yMY03 | y800, PDC1::KanMX-MET3pr-GFP+/- |
| yMY04 | y800, PDC1-ΔTATA::KanMX-MET3pr-GFP+/- |
| yMY05 | y800, CIS1::KanMX-MET3pr-GFP+/- |
| yMY06 | y800, CIS1-ΔTATA::KanMx-MET3pr-GFP+/- |
| yMY07 | y800, CDC20::URA3-GALSpr-mCherry+/-, ATG36::KanMX-GALSpr-GFP+/- |
| yMY08 | y800, CDC20::URA3-GALSpr-mCherry+/-, LDS2::KanMX-GALSpr-GFP+/- |
| yMY09 | y800, CDC20::URA3-GALSpr-mCherry+/-, NTS1::KanMX-GALSpr-GFP+/- |
| yMY10 | y800, CDC20::URA3-GALSpr-mCherry+/-, ALPHA2::KanMX-GALSpr-GFP+/- |
| yMY11 | y800, CDC20::URA3-GALSpr-mCherry+/-, SAM2::KanMX-GALSpr-GFP+/- |
| yMY12 | y800, CDC20::URA3-GALSpr-mCherry+/-, TDH3::KanMX-GALSpr-GFP+/- |
| yMY13 | y800, CDC20::URA3-GALSpr-mCherry+/-, MET13::KanMX-GALSpr-GFP+/- |
| yMY14 | y800, CDC20::URA3-GALSpr-mCherry+/-, RSM23::KanMX-GALSpr-GFP+/- |
| yMY15 | y800, SNT2::KanMX-MET3pr-GFP+/- , CDC20::URA3-MET3pr-mCherry+/- |
| yMY16 | y800, SNT2::KanMX-MET3pr-GFP+/-, MET13::ADE2+/-, CDC20::URA3-MET3pr-mCherry+/- |
| yMY17 | y800, SNT2::KanMX-MET3pr-GFP+/-, MET13::ADE2-/+, CDC20::URA3-MET3pr-mCherry+/- |
| yMY18 | y800, SNT2::KanMX-MET3pr-GFP+/-, MET13::ADE2+/+, LDS2::MET13-LEU2+/-, CDC20::URA3-MET3pr-mCherry+/- |
| yMY19 | y800, RSM23::KanMX-MET3pr-GFP+/-, CDC20::URA3-MET3pr-mCherry+/- |
| yMY20 | y800, CWC23::KanMX-MET3pr-GFP+/-, MET13::ADE2+/-, CDC20::URA3-MET3pr-mCherry+/- |
| yMY21 | y800, CWC23::KanMX-MET3pr-GFP+/-, MET13::ADE2-/+, CDC20::URA3-MET3pr-mCherry+/- |
| yMY22 | y800, CWC23::KanMX-MET3pr-GFP+/-, MET13::ADE2+/+, LDS2::MET13-LEU2+/-, CDC20::URA3-MET3pr-mCherry+/- |
| yMY23 | y800, CWC23::KanMX-MET3pr-GFP+/-, CDC20::URA3-MET3pr-mCherry+/- |
| yMY24 | y800, RSM23::KanMX-MET3pr-GFP+/-, MET13::ADE2+/-, CDC20::URA3-MET3pr-mCherry+/- |
| yMY25 | y800, RSM3::KanMX-MET3pr-GFP+/-, MET13::ADE2-/+, CDC20::URA3-MET3pr-mCherry+/- |
| yMY26 | y800, RSM23::KanMX-MET3pr-GFP+/-, MET13::ADE2+/+, LDS2::MET13-LEU2+/-, CDC20::URA3-MET3pr-mCherry+/- |
| yMY27 | y800, LDS2::CEG1-MET3pr-GFP-MET13-LEU2+/- |
| yMY28 | y800, LDS2::CEG1-MET3pr-GFP-MET13-LEU2+/-, CDC20::URA3-MET3pr-mCherry+/- |
| yMY29 | y800, MET13::MET13-ADE2+/- |
| yMY30 | y800, MET13::MET13-ADE2+/+, LDS2::MET13-LEU2+/- |
| yMY31 | y800, MET13::MET13-ADE2+/-, CDC20::URA3-MET13pr-mCherry+/- |
| yMY32 | y800, MET13::MET13-ADE2+/+, LDS2::MET13-LEU2+/-, CDC20::URA3-MET13pr-mCherry+/- |
