# Supplementary material for: Three distinct mechanisms of long-distance modulation of gene expression in yeast
Source: PLoS Genet. 2017 Apr 20;13(4):e1006736. doi: 10.1371/journal.pgen.1006736 (PMC5417705; doi:10.1371/journal.pgen.1006736)
Supplement: S3 Table — (PPTX) [file pgen.1006736.s010.pptx]

## Slide 1
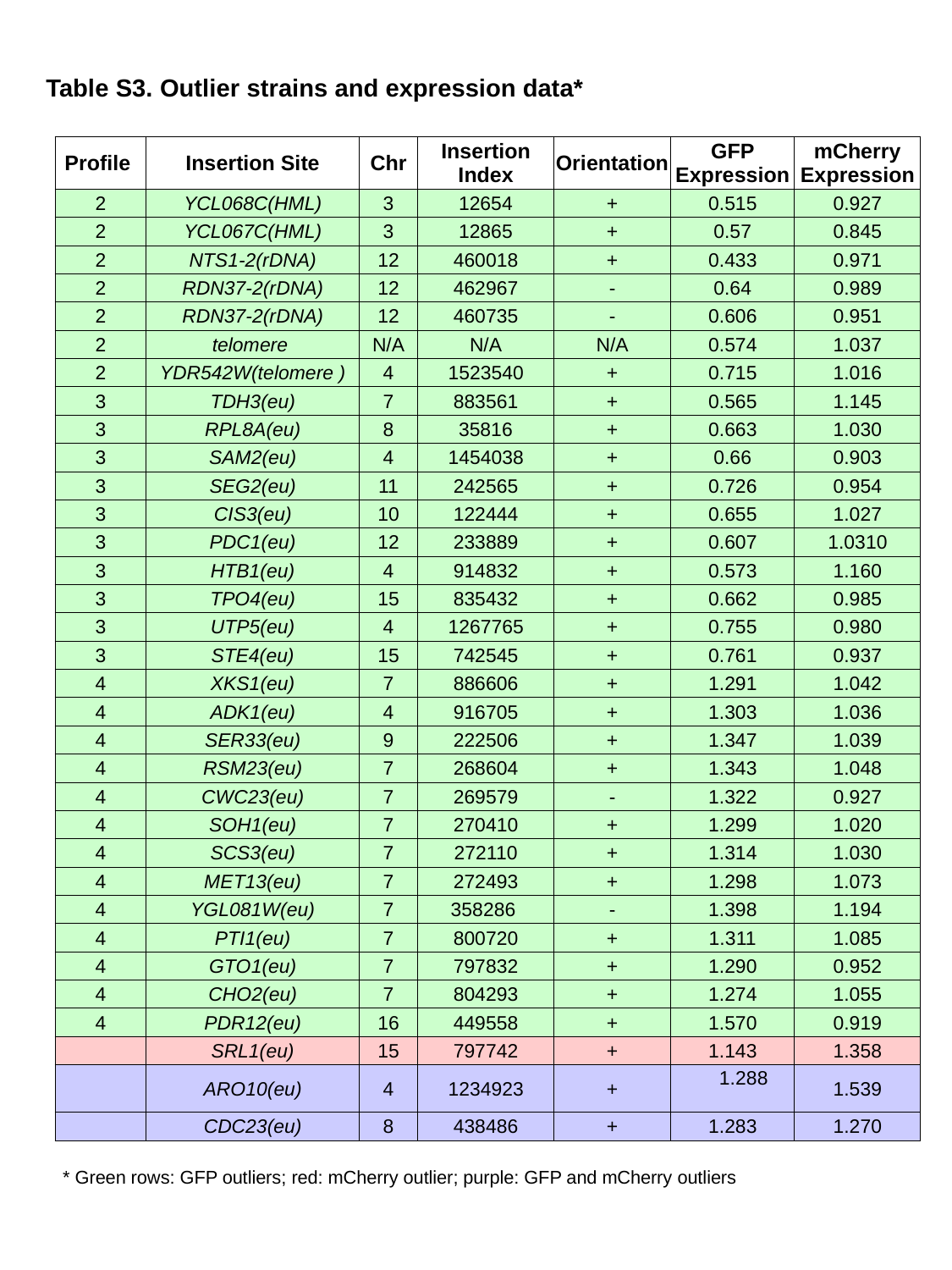

Table S3. Outlier strains and expression data*
| Profile | Insertion Site | Chr | Insertion Index | Orientation | GFP Expression | mCherry Expression |
| --- | --- | --- | --- | --- | --- | --- |
| 2 | YCL068C(HML) | 3 | 12654 | + | 0.515 | 0.927 |
| 2 | YCL067C(HML) | 3 | 12865 | + | 0.57 | 0.845 |
| 2 | NTS1-2(rDNA) | 12 | 460018 | + | 0.433 | 0.971 |
| 2 | RDN37-2(rDNA) | 12 | 462967 | - | 0.64 | 0.989 |
| 2 | RDN37-2(rDNA) | 12 | 460735 | - | 0.606 | 0.951 |
| 2 | telomere | N/A | N/A | N/A | 0.574 | 1.037 |
| 2 | YDR542W(telomere ) | 4 | 1523540 | + | 0.715 | 1.016 |
| 3 | TDH3(eu) | 7 | 883561 | + | 0.565 | 1.145 |
| 3 | RPL8A(eu) | 8 | 35816 | + | 0.663 | 1.030 |
| 3 | SAM2(eu) | 4 | 1454038 | + | 0.66 | 0.903 |
| 3 | SEG2(eu) | 11 | 242565 | + | 0.726 | 0.954 |
| 3 | CIS3(eu) | 10 | 122444 | + | 0.655 | 1.027 |
| 3 | PDC1(eu) | 12 | 233889 | + | 0.607 | 1.0310 |
| 3 | HTB1(eu) | 4 | 914832 | + | 0.573 | 1.160 |
| 3 | TPO4(eu) | 15 | 835432 | + | 0.662 | 0.985 |
| 3 | UTP5(eu) | 4 | 1267765 | + | 0.755 | 0.980 |
| 3 | STE4(eu) | 15 | 742545 | + | 0.761 | 0.937 |
| 4 | XKS1(eu) | 7 | 886606 | + | 1.291 | 1.042 |
| 4 | ADK1(eu) | 4 | 916705 | + | 1.303 | 1.036 |
| 4 | SER33(eu) | 9 | 222506 | + | 1.347 | 1.039 |
| 4 | RSM23(eu) | 7 | 268604 | + | 1.343 | 1.048 |
| 4 | CWC23(eu) | 7 | 269579 | - | 1.322 | 0.927 |
| 4 | SOH1(eu) | 7 | 270410 | + | 1.299 | 1.020 |
| 4 | SCS3(eu) | 7 | 272110 | + | 1.314 | 1.030 |
| 4 | MET13(eu) | 7 | 272493 | + | 1.298 | 1.073 |
| 4 | YGL081W(eu) | 7 | 358286 | - | 1.398 | 1.194 |
| 4 | PTI1(eu) | 7 | 800720 | + | 1.311 | 1.085 |
| 4 | GTO1(eu) | 7 | 797832 | + | 1.290 | 0.952 |
| 4 | CHO2(eu) | 7 | 804293 | + | 1.274 | 1.055 |
| 4 | PDR12(eu) | 16 | 449558 | + | 1.570 | 0.919 |
| | SRL1(eu) | 15 | 797742 | + | 1.143 | 1.358 |
| | ARO10(eu) | 4 | 1234923 | + | 1.288 | 1.539 |
| | CDC23(eu) | 8 | 438486 | + | 1.283 | 1.270 |
* Green rows: GFP outliers; red: mCherry outlier; purple: GFP and mCherry outliers
