# Supplementary material for: Three distinct mechanisms of long-distance modulation of gene expression in yeast
Source: PLoS Genet. 2017 Apr 20;13(4):e1006736. doi: 10.1371/journal.pgen.1006736 (PMC5417705; doi:10.1371/journal.pgen.1006736)
Supplement: S4 Table — (PPTX) [file pgen.1006736.s011.pptx]

## Slide 1
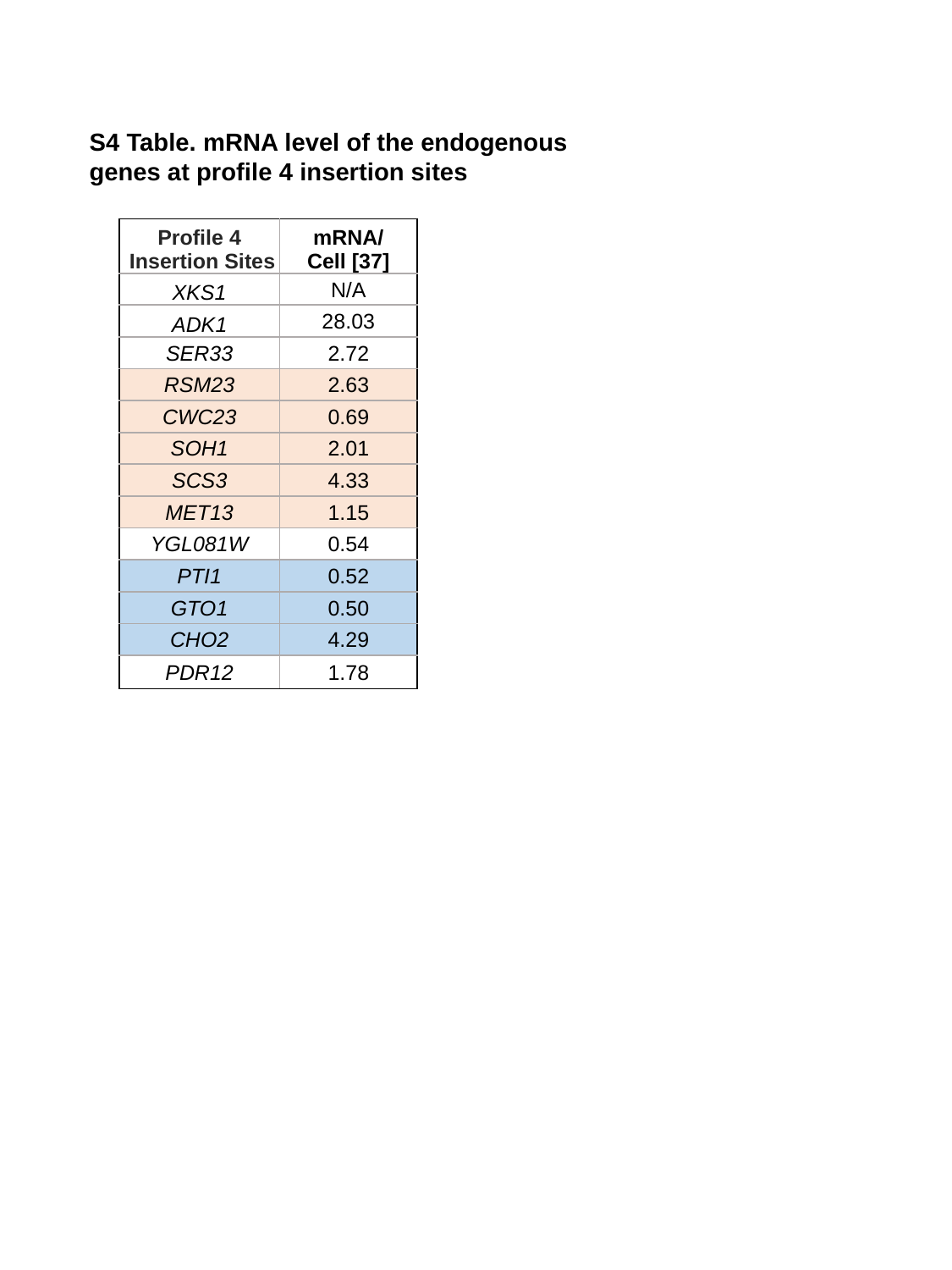

S4 Table. mRNA level of the endogenous genes at profile 4 insertion sites
| Profile 4 Insertion Sites | mRNA/ Cell [37] |
| --- | --- |
| XKS1 | N/A |
| ADK1 | 28.03 |
| SER33 | 2.72 |
| RSM23 | 2.63 |
| CWC23 | 0.69 |
| SOH1 | 2.01 |
| SCS3 | 4.33 |
| MET13 | 1.15 |
| YGL081W | 0.54 |
| PTI1 | 0.52 |
| GTO1 | 0.50 |
| CHO2 | 4.29 |
| PDR12 | 1.78 |
