# Supplementary material for: Three distinct mechanisms of long-distance modulation of gene expression in yeast
Source: PLoS Genet. 2017 Apr 20;13(4):e1006736. doi: 10.1371/journal.pgen.1006736 (PMC5417705; doi:10.1371/journal.pgen.1006736)
Supplement: S5 Table — (PPTX) [file pgen.1006736.s012.pptx]

## Slide 1
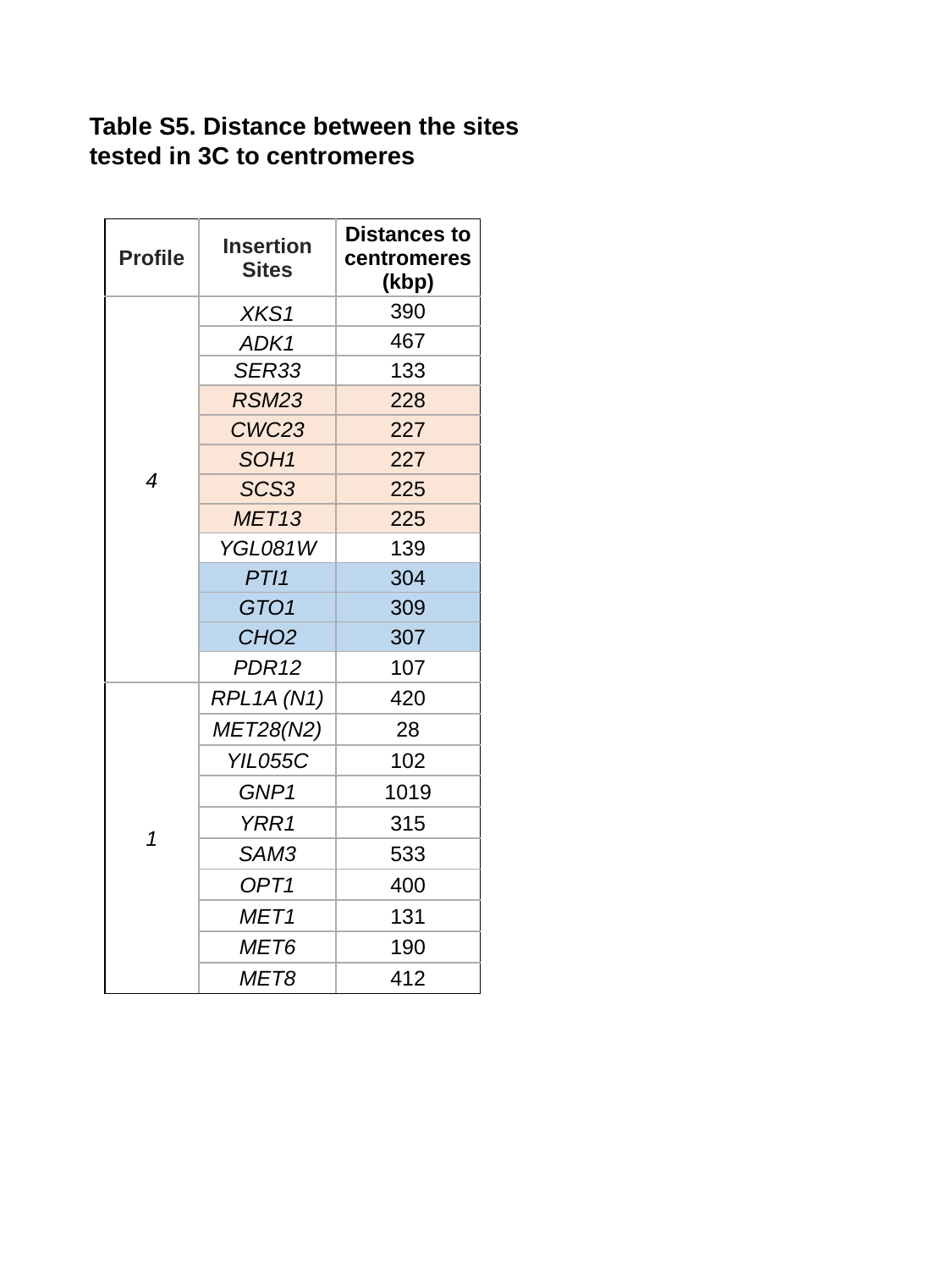

Table S5. Distance between the sites tested in 3C to centromeres
| Profile | Insertion Sites | Distances to centromeres (kbp) |
| --- | --- | --- |
| 4 | XKS1 | 390 |
| | ADK1 | 467 |
| | SER33 | 133 |
| | RSM23 | 228 |
| | CWC23 | 227 |
| | SOH1 | 227 |
| | SCS3 | 225 |
| | MET13 | 225 |
| | YGL081W | 139 |
| | PTI1 | 304 |
| | GTO1 | 309 |
| | CHO2 | 307 |
| | PDR12 | 107 |
| 1 | RPL1A (N1) | 420 |
| | MET28(N2) | 28 |
| | YIL055C | 102 |
| | GNP1 | 1019 |
| | YRR1 | 315 |
| | SAM3 | 533 |
| | OPT1 | 400 |
| | MET1 | 131 |
| | MET6 | 190 |
| | MET8 | 412 |
