# Supplementary material for: Three distinct mechanisms of long-distance modulation of gene expression in yeast
Source: PLoS Genet. 2017 Apr 20;13(4):e1006736. doi: 10.1371/journal.pgen.1006736 (PMC5417705; doi:10.1371/journal.pgen.1006736)
Supplement: S6 Table — (PPTX) [file pgen.1006736.s013.pptx]

## Slide 1
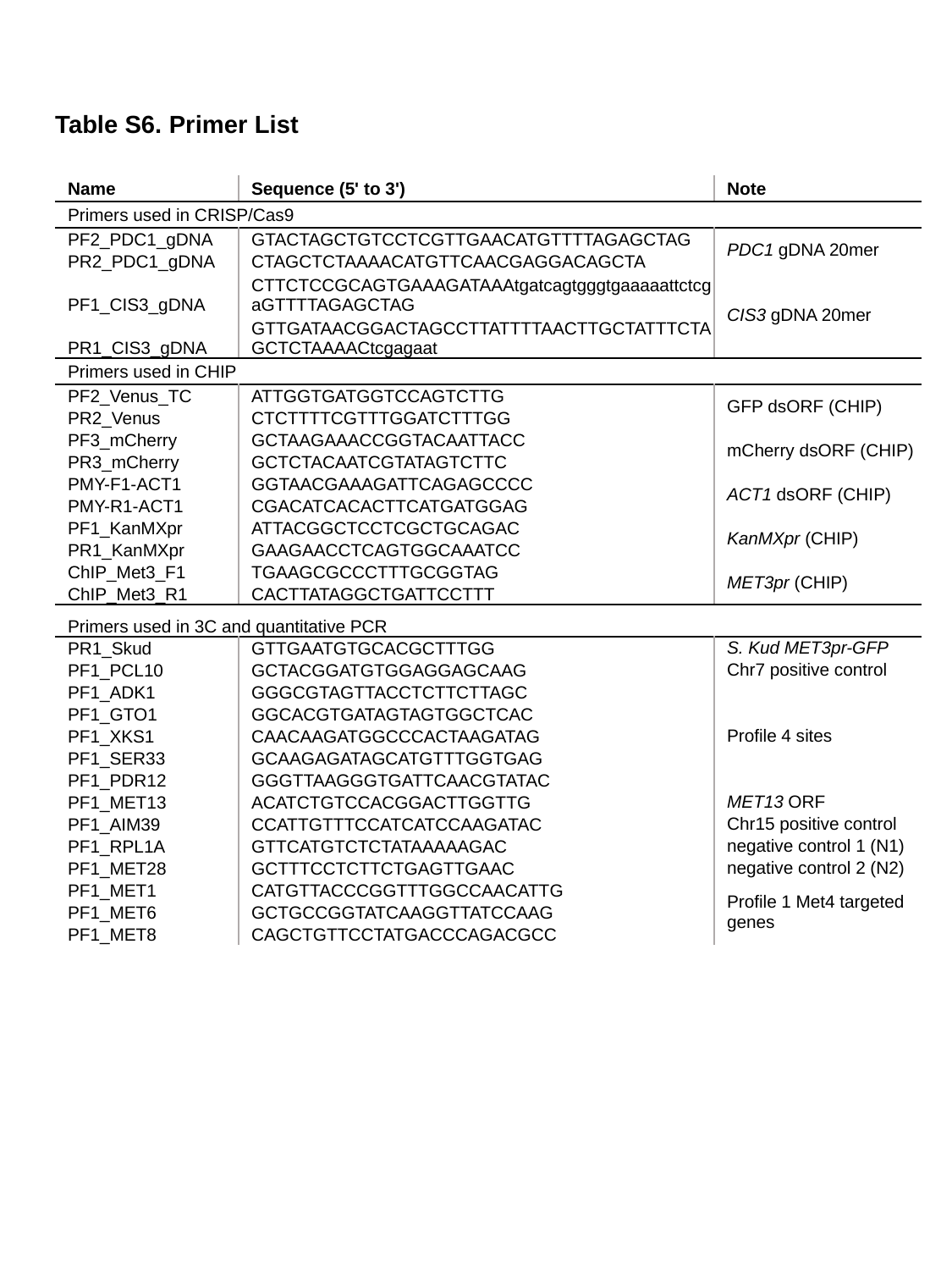

Table S6. Primer List
| Name | Sequence (5' to 3') | Note |
| --- | --- | --- |
| Primers used in CRISP/Cas9 | | |
| PF2\_PDC1\_gDNA | GTACTAGCTGTCCTCGTTGAACATGTTTTAGAGCTAG | PDC1 gDNA 20mer |
| PR2\_PDC1\_gDNA | CTAGCTCTAAAACATGTTCAACGAGGACAGCTA | |
| PF1\_CIS3\_gDNA | CTTCTCCGCAGTGAAAGATAAAtgatcagtgggtgaaaaattctcgaGTTTTAGAGCTAG | CIS3 gDNA 20mer |
| PR1\_CIS3\_gDNA | GTTGATAACGGACTAGCCTTATTTTAACTTGCTATTTCTAGCTCTAAAACtcgagaat | |
| Primers used in CHIP | | |
| PF2\_Venus\_TC | ATTGGTGATGGTCCAGTCTTG | GFP dsORF (CHIP) |
| PR2\_Venus | CTCTTTTCGTTTGGATCTTTGG | |
| PF3\_mCherry | GCTAAGAAACCGGTACAATTACC | mCherry dsORF (CHIP) |
| PR3\_mCherry | GCTCTACAATCGTATAGTCTTC | |
| PMY-F1-ACT1 | GGTAACGAAAGATTCAGAGCCCC | ACT1 dsORF (CHIP) |
| PMY-R1-ACT1 | CGACATCACACTTCATGATGGAG | |
| PF1\_KanMXpr | ATTACGGCTCCTCGCTGCAGAC | KanMXpr (CHIP) |
| PR1\_KanMXpr | GAAGAACCTCAGTGGCAAATCC | |
| ChIP\_Met3\_F1 | TGAAGCGCCCTTTGCGGTAG | MET3pr (CHIP) |
| ChIP\_Met3\_R1 | CACTTATAGGCTGATTCCTTT | |
| Primers used in 3C and quantitative PCR | | |
| PR1\_Skud | GTTGAATGTGCACGCTTTGG | S. Kud MET3pr-GFP |
| PF1\_PCL10 | GCTACGGATGTGGAGGAGCAAG | Chr7 positive control |
| PF1\_ADK1 | GGGCGTAGTTACCTCTTCTTAGC | Profile 4 sites |
| PF1\_GTO1 | GGCACGTGATAGTAGTGGCTCAC | |
| PF1\_XKS1 | CAACAAGATGGCCCACTAAGATAG | |
| PF1\_SER33 | GCAAGAGATAGCATGTTTGGTGAG | |
| PF1\_PDR12 | GGGTTAAGGGTGATTCAACGTATAC | |
| PF1\_MET13 | ACATCTGTCCACGGACTTGGTTG | MET13 ORF |
| PF1\_AIM39 | CCATTGTTTCCATCATCCAAGATAC | Chr15 positive control |
| PF1\_RPL1A | GTTCATGTCTCTATAAAAAGAC | negative control 1 (N1) |
| PF1\_MET28 | GCTTTCCTCTTCTGAGTTGAAC | negative control 2 (N2) |
| PF1\_MET1 | CATGTTACCCGGTTTGGCCAACATTG | Profile 1 Met4 targeted genes |
| PF1\_MET6 | GCTGCCGGTATCAAGGTTATCCAAG | |
| PF1\_MET8 | CAGCTGTTCCTATGACCCAGACGCC | |
